# Multi-objective artificial-intelligence-based parameter tuning of antennas using variable-fidelity machine learning

**DOI:** 10.1038/s41598-025-05657-y

**Published:** 2025-07-01

**Authors:** Slawomir Koziel, Anna Pietrenko-Dabrowska, Stanislaw Szczepanski

**Affiliations:** 1 Engineering Optimization & Modeling Center, Reykjavik University, 102 Reykjavik, Iceland; 2 Faculty of Electronics, Telecommunications and Informatics, Gdansk University of Technology, Gdańsk, 80-233 Poland

**Keywords:** Antenna engineering, Artificial intelligence, variable-fidelity simulations, Surrogate modeling, Artificial intelligence-based design, Machine learning, Neural networks, Electrical and electronic engineering, Computational science

## Abstract

Multi-objective optimization (MO) is an important topic in contemporary antenna design. Due to the reliance on computationally-expensive electromagnetic (EM) simulations, the use of conventional algorithms is prohibitive. These costs can be reduced by appropriate algorithmic tools involving surrogate modeling and soft computing methods. This study introduces an innovative artificial intelligence (AI)-based approach to antenna MO. Our algorithm is a machine learning (ML) procedure employing artificial neural network models. In each iteration, multiple infill vectors are produced, using Pareto ranking of the candidate solution set produced by a multi-objective evolutionary algorithm. The full-wave simulation results acquired for all infill points are incorporated into the dataset to refine the metamodel. Termination of the procedure is based on a comparison of non-dominated solutions obtained in subsequent iterations. Additional reduction of the expenses is enabled through the use of multi-resolution electromagnetic simulations. The presented methodology has been extensively demonstrated with the help of four planar devices, including broadband monopoles and a quasi-Yagi antenna. As shown, the average cost of MO is equivalent to approximately two hundred high-fidelity EM analyses. In absolute terms, 40% of relative speedup is achieved due to variable-fidelity modeling, and almost 90% savings over the one-shot approach. Comparative experiments indicate that the improved computational efficiency of the presented framework is not detrimental to reliability. Consequently, the introduced algorithm can be regarded a feasible alternative to the current MO methodologies for antennas, especially when computational budget is a critical constraint.

## Introduction

Modern antennas are designed to fulfill performance demands imposed by a variety of application areas^1–7^, realize specific functionalities^8–14^, or to conform to strict physical space limitations^15–18^. Meeting stringent requirements leads to topologically involved structures, which often incorporate slots, stubs, metamaterials, defected grounds, etc^19–25^. The complex relationships between design variables and electrical characteristics make meticulous tuning of antenna dimensions imperative, with the process executed using electromagnetic (EM) analysis. A fundamental difficulty of simulation-based design is a significant computational burden whenever conventional algorithms are employed^26,27^. Furthermore, antenna design frequently involves multiple performance objectives, which often stay in conflict^28–30^. Consequently, practical antenna designs invariably involve trade-offs among chosen objectives. Accurate identification of the trade-off design sets necessitates the use of multi-objective optimization (MO)^31^.

Practical EM-driven design still incorporates conventional techniques, primarily based on parameter sweeping^32,33^. However, such methods are only capable of yielding sub-optimal results. Efficient control over several variables, goals, and constraints, requires the application of formal optimization routines^34–37^. Nonetheless, the bulk of existing procedures can only process scalar merit functions, either in local (gradient-based^38^, pattern search^39^) or global search regime^40–43^. Extending these techniques to MO can be achieved by combining objectives^44,45^, or by directly optimizing a selected goal and controlling others through constraints^46^. However, these approaches exhibit a significant bias towards the preferences of the designer concerning the objectives.

Gathering comprehensive information pertaining to potential design trade-offs, commonly identified as a Pareto set^47^, involves multi-objective optimization. This type of information is invaluable for both academic and industrial applications, as it enables a conclusive evaluation of whether an antenna structure is suitable for particular applications. In particular, the designs encapsulated in the Pareto set quantify available trade-offs between the design goals of interest, which facilitates informed decision-making when selecting among diverse options^48–52^. Furthermore, it permits accelerated redesign of the system whenever priorities concerning design goals have been redefined. Nowadays, MO is predominantly executed by means of multi-objective versions of population-based metaheuristic procedures^53–67^. In the realm of simulation-based optimization, the primary drawback pertinent to metaheuristic methods is their suboptimal CPU efficiency. Hence, direct EM-driven MO by nature-inspired techniques is relatively uncommon^68,69^. The associated CPU times vary between several hours and hundreds of hours.

Practical simulation-driven MO can be accomplished by incorporating surrogate modeling techniques^70,71^. The core idea is to perform vast amounts of evaluations of the system under design using a cheap replacement model instead of direct EM analysis. Widely used behavioral modeling techniques in this context include kriging, artificial neural networks, and Gaussian process regression^72–74^. The metamodel might be constructed either up front^75–77^, or during the optimization run^78,79^. Usually, the Pareto set is generated using population-based routines operating on the surrogate model rather than directly on the EM model. The ‘one-shot’ (offline) approaches, where the metamodel is constructed as a separate stage and then optimized, are rare and only applicable for simple cases. Most surrogate-assisted MO algorithms are embedded in machine learning (ML) frameworks. In this process, the metamodel undergoes incremental refinement using full-wave simulation results obtained during the search process. It serves as a predictor to generate candidate designs using various infill criteria^80–82^. Examples of surrogate-assisted algorithms for multi-objective design, also rooted in machine learning frameworks, are detailed in^83–91^; reference^92^ reviews newest ML frameworks for antenna optimization, including the MO algorithms. However, in these approaches, surrogates are built during the optimization process or in the most favorable areas of the design space. This results in a significant portion of the search space being left unexplored. Moreover, dimensionality issues seriously impede surrogate-assisted MO for higher-dimensional cases (with more than 10 variables). Consequently, most of the reported MO procedures are demonstrated using relatively simple test cases.

A possible workaround for dimensionality issues is domain confinement. Therein, the extreme design are identified (i.e., optimizing individual objectives independently), and the domain is defined as the smallest hypercube encapsulating them^29,93,94^, or carrying out a more precise coverage of the Pareto front^95,96^. Reducing the domain’s size decreases the amount of training data required to establish a reliable surrogate model, thus, improves the MO process efficacy. Another option is performance-driven modeling^28,97–100^, where the metamodel is formulated within the region encompassing high-quality vectors w.r.t. the assumed design specifications. This leads to a significant enhancement in model reliability compared to the traditional methods^101–103^. Applying domain confinement paradigm for MO^104–106^ demonstrated favorable outcomes, particularly in enhancing design quality and expediting the design cycle. Nonetheless, domain-confinement approaches come with considerable initial costs (around few hundred EM antenna simulations) of identifying the extreme solutions. Another limitation lies in assumptions related to the set-theoretic connectivity of the Pareto front.

This research introduces a novel artificial intelligence (AI)-based approach to EM-based MO antenna optimization. The process utilizes an artificial neural network (ANN) surrogate serving as a rapid predictor. A number of infill vectors are generated from the Pareto set by optimizing the ANN model using a multi-objective evolutionary algorithm (MOEA) in each iteration of the algorithm. The electromagnetic simulations evaluated at these infill points are incorporated into the training set to refine the surrogate. The termination condition is defined as reaching a satisfactory similarity between electromagnetic-simulated Pareto-optimal designs in consecutive iterations. For additional cost reduction, the proposed algorithm incorporates variable-fidelity models. Initial sampling and ANN model construction are performed at the lowest usable fidelity level. As the algorithm progresses, the fidelity gradually increases, reaching the high-fidelity level after several iterations. We rigorously evaluated our method using four antenna devices, comprising three wideband monopoles designed for size reduction and optimal in-band impedance matching, along with a quasi-Yagi antenna designed for optimal matching and maximal realized gain.

The originality and technical advancements introduced in this work include (i) the development of a machine learning procedure utilizing ANN surrogates and the generation of multiple infill points for multi-objective optimization of antenna structures, (ii) the integration of variable-fidelity EM simulations and the development of a model management scheme to enhance computational efficiency, (iii) the establishment of a robust convergence metric based on the comparison of non-dominated EM-simulated solutions, and (iv) the comprehensive implementation of the entire MO framework, demonstrated across various challenging test cases, coupled with confirming its superiority over various state-of-the-art benchmark techniques.

## Multi-objective antenna optimization by variable-fidelity machine learning

Antennas are transducers converting electric current into electromagnetic waves and the other way around^107^. Their fundamental role is to transmit and receive EM signals. Antennas are essential components in numerous applications including wireless communications, virtual reality devices, wearable/implantable electronics, remote sensing, microwave imaging, satellite communications, EM energy harvesting, and more^108–113^. Antenna design is an intricate endeavor due to the necessity of fulfilling various requirements concerning electrical (e.g., impedance matching) and field performance (radiation pattern, gain, etc.)^114^, while satisfying additional constraints, e.g., limitation of physical size, fabrication costs, etc^115^. Many of the existing objectives are contradictory. Standard design procedures including optimization techniques are typically focused on a single objective. Still, multi-objective optimization renders a much more comprehensive picture of a system’s capabilities by revealing the Pareto front (the set of the best achievable trade-off designs). It allows the designer to choose the right design variation for a given application, which is of fundamental importance, especially from the industrial perspective. Nonetheless, MO of antennas is a challenging undertaking for reasons briefly explained in section “Introduction”, especially high computational costs associated with repetitive EM simulations of the antenna of interest. The issue is particularly pronounced for nature-inspired methods which are nowadays predominantly used for multi-objective and global optimization. In pursuit of expediting the MO process, this paper proposes an alternative approach.

This section delineates the proposed MO optimization procedure, which integrates machine learning, ANN surrogates, and variable-fidelity EM simulations. In this study, multi-objective optimization is treated as synonymous with multi-criterial optimization, and these terms will be used interchangeably. We commence by revisiting the MO task and providing an overview of solution approaches (section “Multi-criterial optimization”). Section “Variable-fidelity computational models” provides a brief discussion of variable-fidelity computational models. Section “Initial sampling. ANN regression models” discusses the details of initial sampling and surrogate modeling using ANNs, while Section “Multi-objective evolutionary algorithm (MOEA)” outlines the specifics of the MOEA procedure employed to generate the Pareto sets. The generation of infill points is outlined in section “Infill point generation”, while the management of variable-fidelity models and the strategy for updating the training dataset are addressed in section “Variable-fidelity model management. Sataset updating strategy”. Section “Termination condition” discusses the termination condition. The operation of the complete algorithm is summarized in section “Proposed algorithm structure and operation” and explained using the flow diagram.

### Multi-criterial optimization

**Fig. 1:**
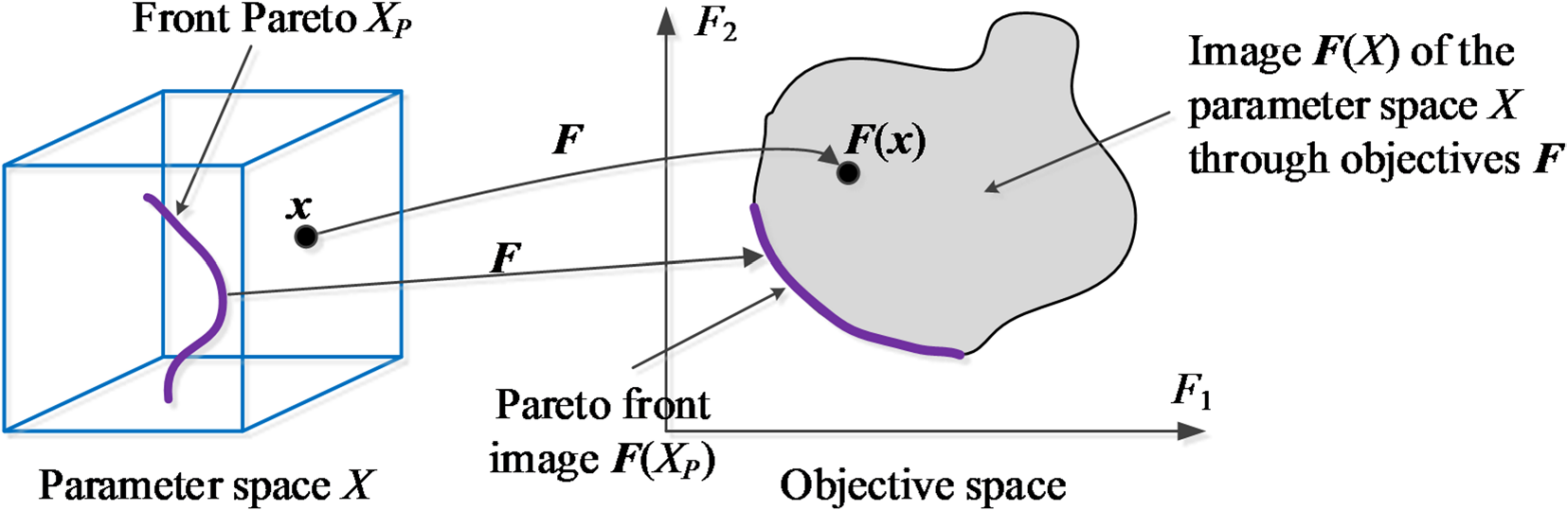
Pareto front for a dual-objective problem (*N*_*obj*_ = 2). The image ***F***(*X*) of the design space *X* is obtained by mapping all parameter vectors ***x*** ∈ *X* using the design goals. The Pareto front *X*_*P*_, marked using a thick line, consists of all designs that are non-dominated in a global sense. Its image ***F***(*X*_*P*_) is the left-bottom part of the boundary of the set ***F***(*X*), also indicated using a thick line in the objective space.

The notation and terminology utilized here in relation to MO optimization has been gathered in Table 1. It should be emphasized that the vector of design variables ***x*** is synonymous with decision variables of the optimization problem. In this study, MO is understood as finding a Pareto set being a discrete representation of the Pareto front denoted as *X*_*P*_. The latter contains all globally non-dominated designs with respect to the relation π^31^, i.e., the best available trade-off designs regarding the considered objectives. Figure 1 presents a graphical illustration. Note that *X*_*P*_ is.

*N*_*obj*_ – 1 (or less) dimensional manifold in the objective space (e.g., a surface for *N*_*obj*_ = 3).

**Table 1:** Notation relevant to multi-objective optimization of antennas.

Symbol	Explanation
***x*** = [*x*_1_ … *x*_*n*_]^*T*^	Vector of design variables
*n*	Number of design variables
*X*	Parameter space defined by the lower and upper bounds on variables ***x***, ***l*** = [*l*_1_ … *l*_*n*_]^*T*^ and ***u*** = [*u*_1_ … *u*_*n*_]^*T*^
***F***(***x***) = [*F*_1_(***x***) *F*_2_(***x***) … *F*_*Nobj*_(***x***)]^*T*^	Vector of design objectives (all to be minimized)
***F***	Objective space
*N*_*obj*_	Number of objectives
*X*_*P*_	Pareto front
π	Pareto dominance relation, defined as follows^31^: design ***x*** dominates over the design ***y*** (or, ***x*** π ***y***) if *F*_*k*_(***x***) ≤ *F*_*k*_(***y***) for all *k* = 1, …, *N*_*obj*_, and *F*_*k*_(***x***) < *F*_*k*_(***y***) for at least one *k*

As already indicated in Sect. 1, MO design optimization can be addressed in different ways. Perhaps the simplest approach is objective aggregation, as it enables the employment of single-objective algorithms. The most common example is a weighted sum method^44^, where the scalar objective is defined as

1 $$\sum\nolimits_{{k=1}}^{{{N_{obj}}}} {{a_k}{F_k}({\boldsymbol{x}})} ,\;\;\;\;\;{a_k}>0,\;\;\;i=1,2,...,{N_{obj}},\;\;\;\;\;\;\sum\nolimits_{{k=1}}^{{{N_{obj}}}} {{a_k}=1}$$

where *a*_*k*_, *k* = 1, …, *N*_*obj*_ denote weights. Clearly, solving (1) only allows us to find a single Pareto-optimal point. Obtaining the entire Pareto set requires repeating the optimization process with different weight arrangements. Yet, obtaining a set of uniformly-distributed solutions is difficult. Also, non-convex sections of the front are not attainable (which can be addressed by, e.g., a goal attainment method^116^).

Nowadays, MO problems are typically solved using population-based algorithms^54–67^, which can produce the complete Pareto set during a single optimization run. However, direct EM-driven MO of antennas using nature-inspired methods is rarely an option due to excessive computational cost. Instead, surrogate-assisted methods are employed, often embedded in machine learning frameworks (cf^83–92^). Unfortunately, difficulties in constructing reliable surrogate models over larger design spaces (higher dimensionality, wide ranges of geometry parameters) constitute the major bottleneck of these techniques. The introduced methodology aims to address some of these issues by combining machine learning, multiple infill point generation, and variable-fidelity EM simulations. The individual parts of the procedure are discussed in the subsequent sections.

### Variable-fidelity computational models

EM-driven design of antennas is typically carried out with a single type of computational model, which is a high-fidelity (or fine) one ***R***_*f*_(***x***). The model is selected to ensure adequate accuracy and configured so that increasing the discretization density of the structure does not cause significant alterations in the represented characteristics. Normally, ***R***_*f*_(***x***) also ensures a satisfactory alignment between the EM-evaluated and measured outputs of the antenna prototype. Unfortunately, evaluating ***R***_*f*_(***x***) is costly, especially for geometrically intricate structures (comprising metamaterial cells, shorting pins, substrate-integrated waveguides) or components such as the SMA connectors, etc.

Accelerating the simulation process is possible by reducing the model fidelity, typically by using coarser discretization of the structure^117^. The low-fidelity (or coarse) representation ***R***_*c*_(***x***) is faster but of limited accuracy. In most cases, it has to be enhanced to become a reliable predictor^118–120^ Still, raw ***R***_*c*_(***x***) may be utilized for certain purposes, e.g., design space pre-screening^121,122^. Normally, two levels of fidelity are employed. Recently, the potential benefits of employing a continuous spectrum of resolutions have proven^123^.

**Fig. 2:**
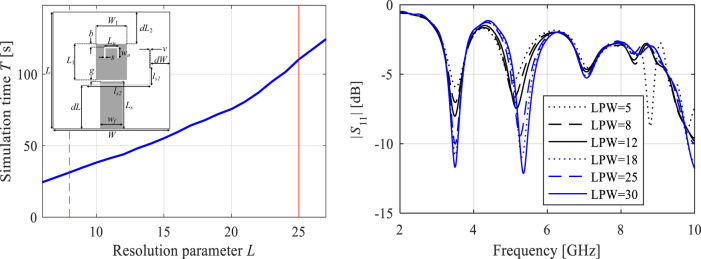
Multi-resolution simulations for an exemplary triple-band microstrip antenna. Shown are: parameterized antenna geometry, reflection responses for different discretization densities (as set by the LPW parameter), and EM simulation time as a function of LPW. LPW values representing high-fidelity (—) and low-fidelity model (- - -) are marked using vertical lines.

Figure 2 demonstrates the typical relationship between model fidelity, the accuracy of antenna response representation, and simulation time for a triple-band microstrip antenna. The model resolution *L* is altered by setting the lines per wavelength (LPW) coefficient in CST Microwave Studio. The minimum usable fidelity, denoted as *L*_min_, is configured to avoid significant distortion of antenna characteristics with any further reduction (approximately 8 in this case). The maximum fidelity, *L*_max_, is established through a grid convergence study to ensure that further increases in LPW do not result in noticeable changes to the antenna responses (around 25).

**Table 2:** Notation relevant to variable-fidelity computational models.

Symbol	Explanation
*L*	Model fidelity
*L*_*min*_	Minimum usable fidelity (corresponding to coarse model)
*L*_*max*_	Maximum fidelity (corresponding to fine model)
***R***(***x***)	Response of the EM simulation model
***R***_*c*_(***x***) = ***R***(***x***,*L*_min_)	Response of the coarse simulation model
***R***_*f*_(***x***) = ***R***(***x***,*L*_max_)	Response of the fine simulation model

In this investigation, search process is expedited using multi-fidelity EM analysis. We apply a continuous spectrum of fidelity *L* from *L*_min_ to *L*_max_. The generic EM model of resolution *L* is referred to as ***R***(***x***,*L*); in particular, ***R***_*f*_(***x***) = ***R***(***x***,*L*_max_), and ***R***_*c*_(***x***) = ***R***(***x***,*L*_min_). The notation related to variable-resolution EM simulations is summarized in Table 2. The details concerning model management will be provided in the later sections.

### Initial sampling. ANN regression models

The proposed MO procedure is a surrogate-assisted algorithm employing ANN regression models. The first stage is to allocate *N*_*init*_ random samples within the parameter space *X* using a modified Latin Hypercube Sampling (LHS) scheme^124^. *N*_*init*_ is a control parameter of the algorithm to be discussed later. The data points are denoted as ***x***_*B*_^(*j*)^, *j* = 1, …, *N*_*init*_. EM analysis is performed at each of these points to acquire antenna characteristics ***R***(***x***,*L*_min_), i.e., the initial dataset is prepared with the use of the least accurate EM model.

**Fig. 3:**
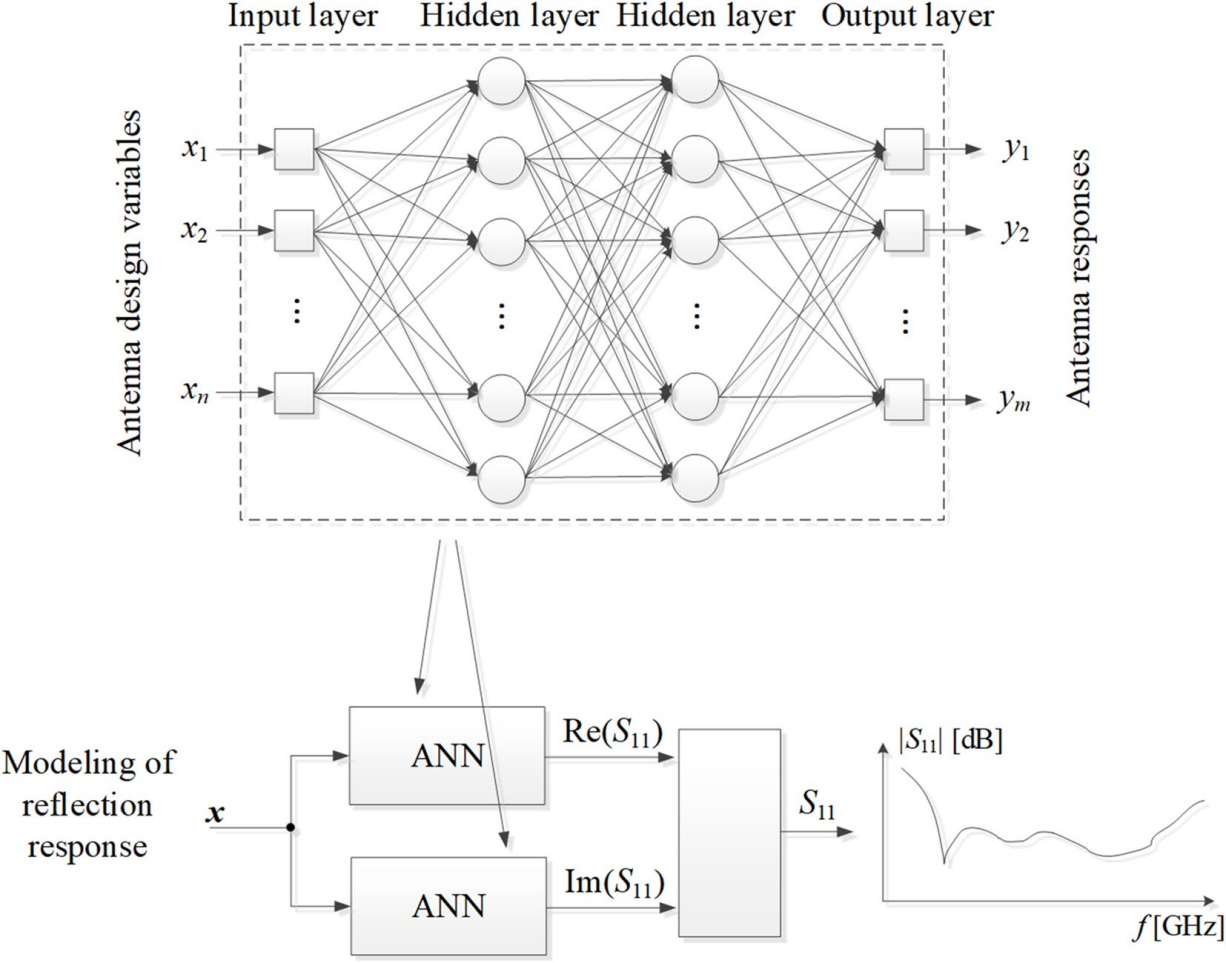
The architecture of the ANN surrogate model utilized in this study, which is a multi-layer perceptron. In the case of complex responses (e.g., reflection response shown in the picture), separate models are generated for real and imaginary parts, which are merged afterwards. The outputs of ANN model are antenna responses at consecutive frequencies *f*_1_ through *f*_*m*_.

The ANN surrogate employed in this study is a multi-layer perceptron^125,126^ featuring two hidden layers, each containing ten neurons. It utilizes a sigmoid activation function and employs a gradient-based algorithm^127^ for network learning process (performance metric: mean squared error, random between training/testing data, and maximum of 1000 learning epochs). The number of hidden layers and their size have been experimentally determined to prove sufficient for the size of the considered test problems (search space dimensionality around *n* = 10). The ANN model’s inputs are antenna design variables (vector ***x***), whereas the outputs are frequency characteristics (e.g., reflection characteristics |*S*_11_| or realized gain vs. frequency). For complex-valued responses, such as *S*_11_, real and imaginary parts are modelled independently. It should also be noted that due to the simple structure of the neural network, its learning is fast (typically, between ten seconds and one minute), so the computational costs of model rendition can essentially be considered meaningless as compared to EM analysis of the antenna of interest. A generic architecture of the ANN utilized here has been shown in Fig. 3.

A comment should be made regarding the selection of the surrogate model. During multi-objective optimization, the surrogate model constructed in the current iteration of the search process is used to predict the location of the updated Pareto set, which is partly associated with extrapolation of data (the new Pareto optimal designs are typically located slightly outside the current approximation of the optimal set). Consequently, we need a relatively simple regression model, which balances approximation and extrapolation capability. A simple neural network, such as the one employed in this study, fulfils this prerequisite. Kernel-based models such as GPR, radial basis functions (RBF), or kriging is not as good in this respect. In particular, RBF or kriging are interpolative models that are poor in terms of extrapolation. The same can be said about more complex neural network surrogates. Our initial experiments indicated that using a straightforward multilayer perceptron with just two hidden layers is sufficient.

In terms of approximation capability, the predictive power of the ANN surrogate utilized in this work is comparable to other types of regression models (GPR, SVR, RBF, kriging). The initial experiments indicate that the relative root-mean-squared error (RRMSE) is typically between five to ten% for the test antenna structures considered in Sect. 3, assuming 100-sample training set allocated along the Pareto front. However, as mentioned earlier, extrapolation capability of these surrogates is considerably worse.

### Multi-objective evolutionary algorithm (MOEA)

In each iteration of the multi-objective process, a rendition of the Pareto front is generated by optimizing the ANN metamodel in a multi-criterial sense. The core algorithm employed here is a multi-objective evolutionary algorithm with a floating-point representation. The algorithm setup includes: a generational model (where the new population entirely replaces the previous one), Pareto-ranking-based tournament selection, fitness sharing with adaptively adjusted niche size, multi-point elitism, and a termination condition based on a sufficient reduction of newly created Pareto-optimal solutions. The algorithm’s implementation follows the guidelines discussed in^128^. The recombination operator is a combination of an arithmetic and intermediate crossover (with even likelihood)^129^.

Let’s denote parental solutions as ***x*** = [*x*_1_ … *x*_*n*_]^*T*^ and ***y*** = [*y*_1_ … *y*_*n*_]^*T*^, and descendant solutions as ***z*** = [*z*_1_ … *z*_*n*_]^*T*^, the intermediate crossover yields the vector ***z*** such that *z*_*i*_ = *ax*_*i*_ + (1-*a*)*y*_*i*_ with 0 ≤ *a* ≤ 1 (a selected randomly). Whereas the arithmetic crossover produces ***z*** = *a****x*** + (1 – *a*)***y*** with 0 ≤ *a* ≤ 1 (*a* selected randomly). The mutation introduces localized random perturbations, independently for each antenna parameter. We have *x*_*i*_ → *x*_*i*_’ = *x*_*i*_ + Δ*x*_*i*_, with random deviation Δ*x*_*i*_^129^

2 $$\Delta {x_i}=\left\{ \begin{gathered} \left( {{x_{i.\hbox{max} }} - {x_i}} \right) \cdot {\left( {2(r - 0.5)} \right)^\beta }\;\;{\text{if}}\;\;r>0.5 \hfill \\ \left( {{x_{i.\hbox{min} }} - {x_i}} \right) \cdot {\left( {2(0.5 - r)} \right)^\beta }\;\;{\text{otherwise}} \hfill \\ \end{gathered} \right.$$

where *r* ∈ [0,1] is a random number, *β* = 3, whereas *x*_*i*.min_ and *x*_*i*.max_ refer to the lower and upper bound, respectively.

In the experiments of Sect. 3, the population size is configured as *N*_*P*_ = 200, with crossover probability set to *p*_*m*_ = 0.8 and mutation probability equal to *p*_*c*_ = 0.1. It is crucial to note that since the MOEA algorithm works with the fast metamodel. Thus, the CPU cost of generating the Pareto set can be disregarded in comparison to even an individual antenna EM simulation.

### Infill point generation

The infill points are the candidate solutions generated iteration-wise during the machine-learning-based MO procedure, and incorporated into the overall training set. These designs are chosen from the present Pareto set created by the MOEA procedure via ANN model optimization. The prerequisite is to cover the Pareto front as uniformly as possible. Therefore, the infill points as close as possible to the target levels *F*_*j*_ within the admissible range of *F*_2_ are selected. The latter is decided upon by the Pareto front span as well as optional additional conditions (e.g., − 10 dB admittance limit for maximum in-band reflection if the first objective if associated with impedance matching). The intended values are: *F*_*j*_ = *F*_2.min_ + ∆*F*⋅(*j* – 1)/(*N*_*infill*_ – 1), where ∆*F* = *F*_2.max_ – *F*_2.min_, *N*_*infill*_ is the infill point number (a control parameter of the procedure). The infill vectors generated in iteration *i* of the multi-objective optimization run will be denoted as ***x***_*I*_^(*i.j*)^, *j* = 1, …, *N*_*infill*_.

Even though the infill points are chosen from the MOEA-rendered Pareto set, the values of the design objectives computed from EM-simulated antenna characteristics are typically inferior (i.e., not Pareto-optimal) due to the limited predictive power of the ANN model. In essence, the metamodel is regarded as a supplementary tool in the machine learning process. Ultimately, the only relevant information that matters, both in terms of design objective values, Pareto set, and termination condition of the search process, is the EM-simulated data.

### Variable-fidelity model management. Dataset updating strategy

As mentioned earlier, the proposed algorithm employs variable-fidelity EM models. The fidelity *L* varies between the lowest level *L*_min_ and the highest one *L*_max_, as elaborated on in Sect. 2.2. Initial sampling is entirely executed using *L*_min_, which enables considerable computational savings. However, during the MO process, the model fidelity has to be gradually increased, to eventually reach *L*_max_, thereby ensuring the reliability of evaluated antenna characteristics and the Pareto-optimal designs.

Here, a simple model management strategy is introduced, according to which the resolution *L*^(*i*)^ of EM analysis in the *i*th iteration equals

3 $${L^{(i)}}=\hbox{min} \left\{ {{L_{\hbox{max} }},{L_{\hbox{min} }}+({L_{\hbox{max} }} - {L_{\hbox{min} }})\frac{{i - 1}}{{i - {N_{transition}}}}} \right\}$$

where *N*_*transition*_ (a control parameter) is the number of iterations after which model fidelity attains the maximum level of *L*_max_.

An important component of the algorithm is the strategy for updating the dataset used to construct the ANN surrogate. In single-fidelity approach, all EM-simulated data samples, including the initial set of samples ***x***_*B*_^(*j*)^, *j* = 1, …, *N*_*init*_, and all infill points acquired up to iteration *i* inclusive, i.e., ***x***_*I*_^(*k.j*)^, *k* = 1, …, *i*, and *j* = 1, …, *N*_*infill*_, would be concatenated to form the overall dataset. However, in a variable-fidelity regime, the less accurate samples have to be systematically withdrawn from the set, ensuring that only high-fidelity data samples remain in the end. Below, the specific strategy for updating dataset has been described.

Let ***x***_*T*_^(*i*–1.*j*)^, *j* = 1, …, *N*_*T*_, be the current dataset (available at the end of the *i*th iteration of the MO process), and ***R***(***x***_*T*_^(*i*–1.*j*)^,*L*^(*i*–1.*j*)^) be the corresponding EM-simulated antenna characteristics. For example, after initial sampling (before the first iteration), we have the current number of samples *N*_*T*_ = *N*_*init*_, ***x***_*T*_^(0.*j*)^ = ***x***_*B*_^(*j*)^, *j* = 1, …, *N*_*T*_, and *L*^(0.*j*)^ = *L*_min_ for *j* = 1, …, *N*_*T*_ (cf. Section 2.3). Further, let ***x***_*I*_^(*i.j*)^, *j* = 1, …, *N*_*infill*_, be the infill samples generated in the *i*th iteration, and ***R***(***x***_*I*_^(*i.j*)^,*L*^(*i.j*)^) be the corresponding EM-simulated antenna responses. The updated dataset {***x***_*T*_^(*i*.*j*)^} is constructed using the following rules:

- If *N*_*T*_ + *N*_*infill*_ ≤ 2*N*_*init*_, then the updated dataset is obtained by concatenating the current data set {***x***_*T*_^(*j*)^} and the infill samples {***x***_*I*_^(*i.j*)^}, i.e., {***x***_*T*_^(*i.j*)^} = {***x***_*T*_^(*i*–1.*j*)^} ∪ {***x***_*I*_^(*i.j*)^}, as well as the respective antenna responses;
- If *N*_*T*_ + *N*_*infill*_ > 2*N*_*init*_, we consider the following options:
  - If the concatenated set {***x***_*T*_^(*i*–1.*j*)^} ∪ {***x***_*I*_^(*i.j*)^} contains the EM-evaluated data obtained at the fidelity level *L* < *L*_max_, then remove *N*_*T*_ + *N*_*infill*_ – 2*N*_*init*_ points evaluated at the (currently) lowest fidelity level;
  - If the concatenated set {***x***_*T*_^(*i*–1.*j*)^} ∪ {***x***_*I*_^(*i.j*)^} only contains the EM-evaluated data EM simulation data obtained at the fidelity level *L* = *L*_max_, then take {***x***_*T*_^(*i.j*)^} = {***x***_*T*_^(*i*–1.*j*)^} ∪ {***x***_*I*_^(*i.j*)^} as the updated set.

This strategy allows for maintaining the training set of the size up to 2*N*_*infill*_ until it contains EM-simulated antenna characteristics evaluated at the fidelity level lower than *L*_max_. The currently lowest-fidelity points are gradually removed from the set until all samples therein are high-fidelity ones. Subsequently, the dataset expands, as only high-fidelity points (*L* = *L*_max_) are generated by the machine learning process.

**Fig. 4:**
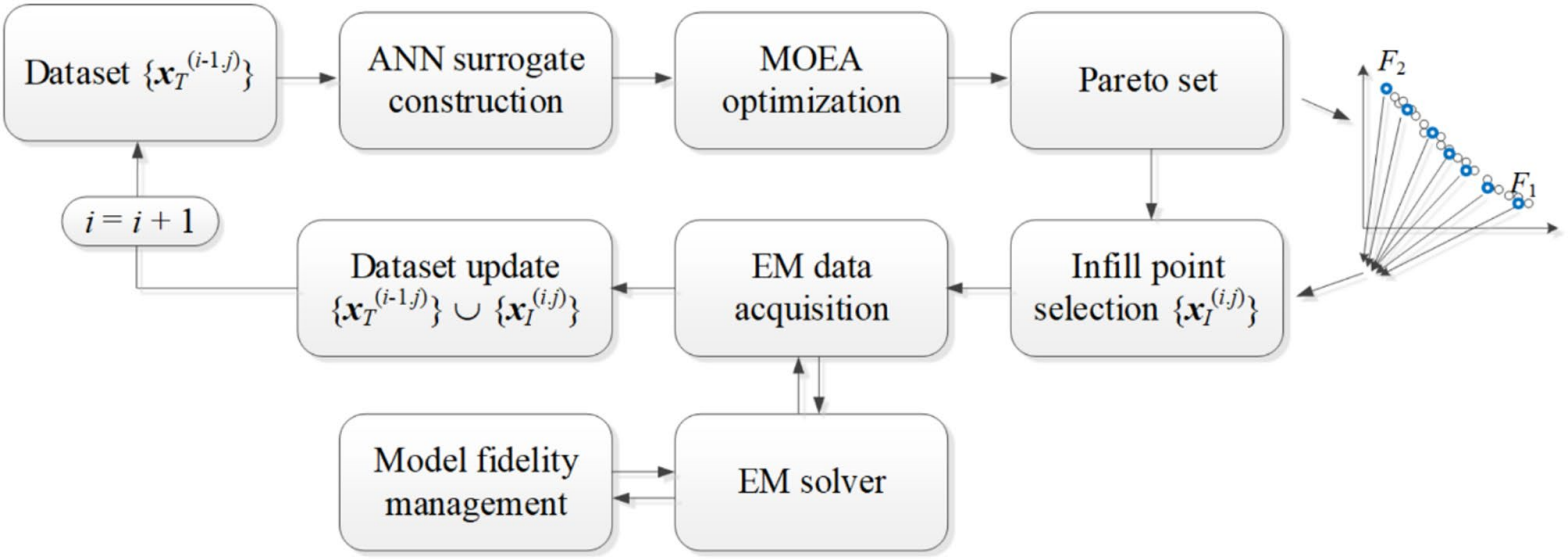
Workflow of the machine learning loop. Pareto set is generated using MOEA by optimizing ANN model built based on the current set of samples {***x***_*T*_^(*i*–1.*j*)^}. Next, the selection of the infill points is carried out and the respective EM simulations data are evaluated using the recent fidelity. The dataset update concludes the iteration.

Figure 4 illustrates the flow diagram depicting the process of constructing the ANN model using the current database, generating the Pareto set through MOEA optimization, selecting infill points, and updating the dataset.

### Termination condition

**Fig. 5:**
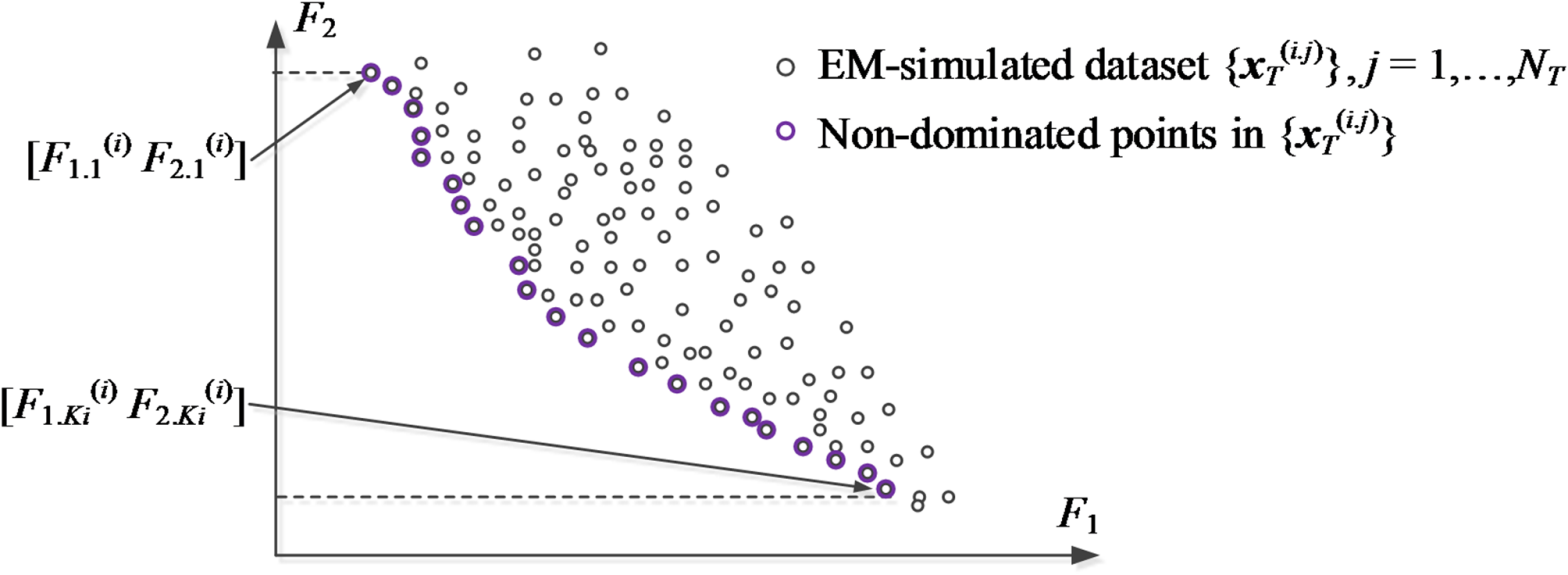
Current (*i*th iteration) EM-evaluated dataset {***x***_*T*_^(*i.j*)^}: small circles mark *N*_*T*_ samples and large circles indicate the Pareto non-dominated solutions selected therefrom, with [*F*_1.*j*_^(*i*)^ *F*_2.*j*_^(*i*)^], *j* = 1, …, *K*_*i*_, where *K*_*i*_ is the number of non-dominated points, being the corresponding set of design objectives.

The machine learning MO process is continued until convergence. Convergence of the (iterative) optimization process is interpreted as a situation when the candidate designs produced in subsequent iterations become sufficiently close to each other. In other words, if the algorithm does not further advance towards the optimum design (in the multi-objective context, it does not further improve the Pareto set). When this happens, the process is terminated as additional enhancements are below the prescribed threshold. The following description provides the details of the convergence metric employed in this study. As mentioned earlier, we are only interested in EM-simulated data points, meaning that the Pareto set similarity is considered at this level. Figure 5 visualizes the current dataset {***x***_*T*_^(*i.j*)^}_*j*=1, …, *Ki*_, as well as its Pareto non-dominated part with the corresponding objective vectors [*F*_1.*j*_^(*i*)^ *F*_2.*j*_^(*i*)^]. We assume that the points are arranged in ascending order w.r.t. *F*_1_, i.e., *F*_1.*j*_^(*i*)^ ≤ *F*_1.*k*_^(*i*)^ for *j* < *k*. Further, let ***F***_*nondom*_^(*i*)^ be a *M* × 2 matrix

4 $${\boldsymbol{F}}_{{nondom}}^{{(i)}}=\left[ {\begin{array}{*{20}{c}} {F_{{I.1.1}}^{{(i)}}}&{F_{{I.2.1}}^{{(i)}}} \\ {F_{{I.1.2}}^{{(i)}}}&{F_{{I.2.2}}^{{(i)}}} \\ \vdots & \vdots \\ {F_{{I.1.M}}^{{(i)}}}&{F_{{I.2.M}}^{{(i)}}} \end{array}} \right]$$

with

5 $$F_{{I.1.k}}^{{(i)}}=F_{{1.1}}^{{(i)}}+\left[ {F_{{1.{K_i}}}^{{(i)}} - F_{{1.1}}^{{(i)}}} \right]\frac{{k - 1}}{{M - 1}}$$

and

6 $$F_{{I.2.k}}^{{(i)}}=I\left( {F_{{I.1.k}}^{{(i)}},\left[ {F_{{1.1}}^{{(i)}}\;...\;F_{{1.{K_i}}}^{{(i)}}} \right],\left[ {F_{{2.1}}^{{(i)}}\;...\;F_{{2.{K_i}}}^{{(i)}}} \right]} \right)$$

where *I*(*y*,[*y*_1_ … *y*_*K*_], [*z*_1_ … *z*_*K*_]) is a function interpolating the value vector [*z*_1_ … *z*_*K*_] corresponding to the parameter vector [*y*_1_ … *y*_*K*_] on the input parameter *y*. Here, *M* is a large integer, e.g., 100. The operation (4)-(6) interpolates the set {*F*_1.*j*_^(*i*)^ *F*_2.*j*_^(*i*)^} onto a matrix of a fixed size *M* × 1 (regardless of the specific number of non-dominated points *K*_*i*_ in iteration *i*), thereby enabling comparison of the Pareto sets between subsequent iterations.

Using the definition of ***F***_*nondom*_^(*i*)^, we can now define a similarity metric *E*_*i*_, which is defined for *i* = 1, 2, …, as

7 $${E_i}=\left\| {{\boldsymbol{F}}_{{nondom}}^{{(i)}} - {\boldsymbol{F}}_{{nondom}}^{{(i - 1)}}} \right\|$$

We also define a moving average *E*_*a.i*_ as.

8 $${E_{a.i}}=\frac{1}{{i - \hbox{max} \left\{ {1,i - {N_a}+1} \right\}+1}}\sum\limits_{{k=\hbox{max} \{ 1,i - {N_a}+1\} }}^{i} {{E_k}}$$

The moving average *E*_*a.i*_ smooths out possible fluctuations in the similarity metric *E*_*i*_ caused by the stochastic components in the search process.

The termination criterion for the MO process is defined using *E*_*a.i*_ as follows:

9 $${E_{a.i}}<\varepsilon$$

where *ε* is the convergence threshold (a control parameter of the algorithm).

### Proposed algorithm structure and operation

This section summarizes the details of the developed variable-fidelity MO optimization algorithm. We start by discussing the control parameters of the algorithm, which have been gathered in Table 3. There are only a few parameters, mainly concerning the dataset sizes and the iteration span for EM model fidelity transition as well as moving average computation. The baseline values of Table 3, will be utilized in all verification experiments detailed in Sect. 3. Additionally, it is worth noting that the configuration of the MOEA algorithm is established as discussed in Sect. 2.4. The specific values of its control parameters are of minor significance since MOEA optimizes a fast ANN surrogate. Therefore, it can be executed with an ample computational budget without significantly impacting the expenses related to MO process. The latter essentially stem from the number of the performed EM evaluations.

**Fig. 6:**
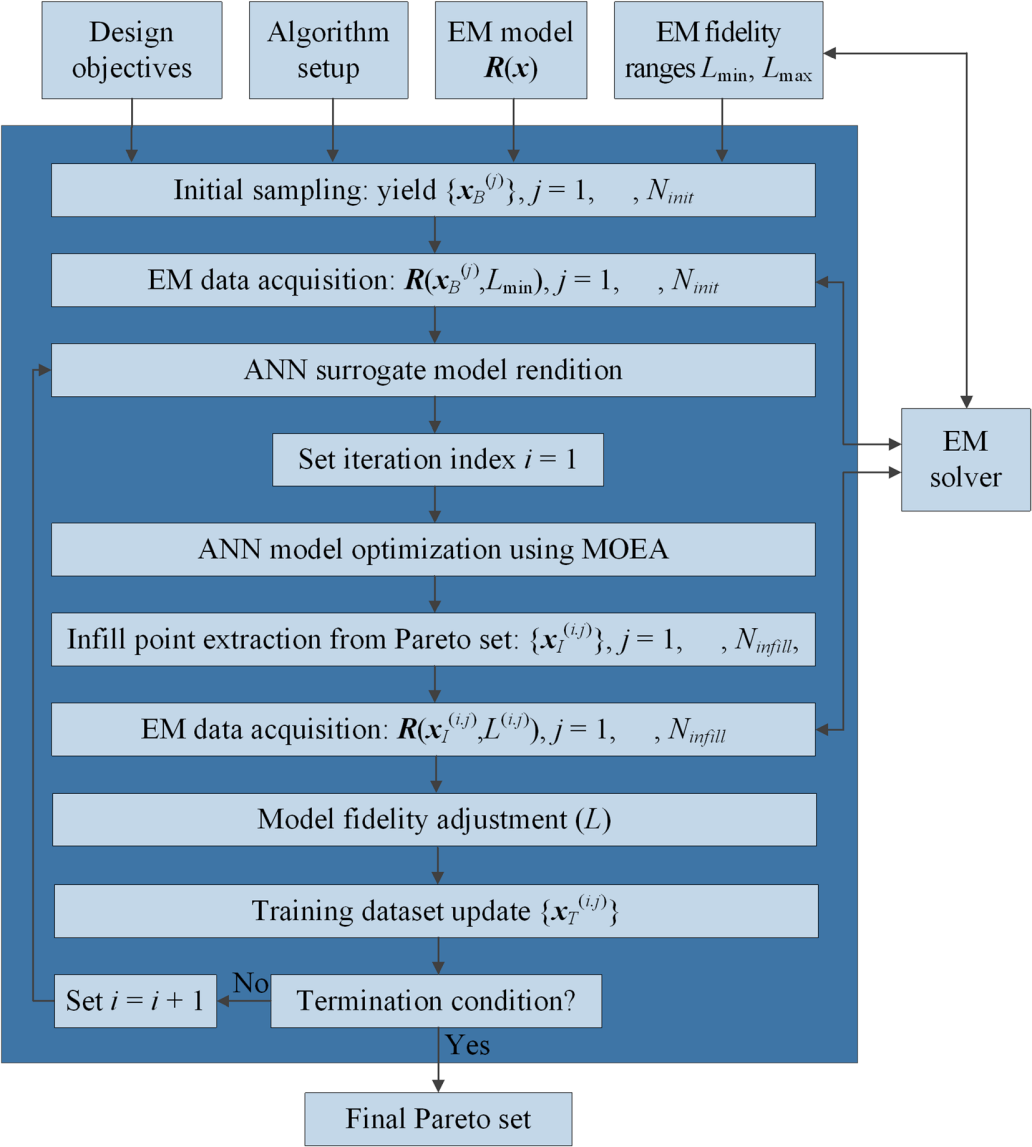
Flow diagram of the proposed multi-objective optimization procedure with machine learning and variable-fidelity EM models.

Figure 6 illustrates the flow of our multi-objective optimization framework. The main steps of the MO process include: (i) preliminary data collection and acquiring of the EM data, (ii) construction of the ANN surrogate model, (iii) rendition of the Pareto-optimal designs using MOEA, (iv) extraction of the infill points and EM-data collection, as well as (v) adjusting the EM model fidelity.

**Table 3:** MO algorithm with machine learning and variable-fidelity EM models: control parameters.

Parameter	Description	Default value
*N*_*init*_	The number of initial sample points for constructing the first ANN surrogate (cf. Sect. 2.3)	100
*N*_*infill*_	The number of infill points generated per MO iteration (cf. Section 2.5)	10
*N*_*transition*_	The number of iterations over which the EM model fidelity is changed from *L*_min_ to *L*_max_ (cf. Section 2.6)	5
*N*_*a*_	The span of the moving average for computing the Pareto front similarity metric *E*_*a.i*_ (cf. Section 2.7)	5
*ε*	Termination threshold for the MO process (cf. Section 2.7)	1

It should be reiterated that the preliminary sampling is carried out at the coarsest fidelity, and the fidelity level increases to reach *L*_max_ during the first few iterations of the ML algorithm. At the same time, the less accurate data is gradually withdrawn from the dataset, which allows for maintaining reliability. Adding infill points near the predicted Pareto front helps focus on that area and improves the ANN model’s accuracy there. This eventually leads to convergence and yielding a reliable representation of the Pareto front (as evaluated using EM analysis).

## Demonstration case studies

This section depicts the proposed variable-fidelity machine learning approach for low-cost antenna multi-objective optimization using four microstrip structures. These structures include three wideband miniaturized monopoles and a quasi-Yagi antenna. We also compare our framework to several surrogate-assisted MO optimization techniques. Our experiments aim to validate the computational efficiency and reliability of the algorithm, as well as to assess its potential advantages over state-of-the-art benchmark routines. The content of this section is divided into several parts: Sect. 3.1 introduces the test problems, Sect. 3.2 describes the experimental setup and outlines the benchmark techniques, Sect. 3.3 presents the results, and Sect. 3.4 summarizes the findings.

### Verification case studies

**Fig. 7:**
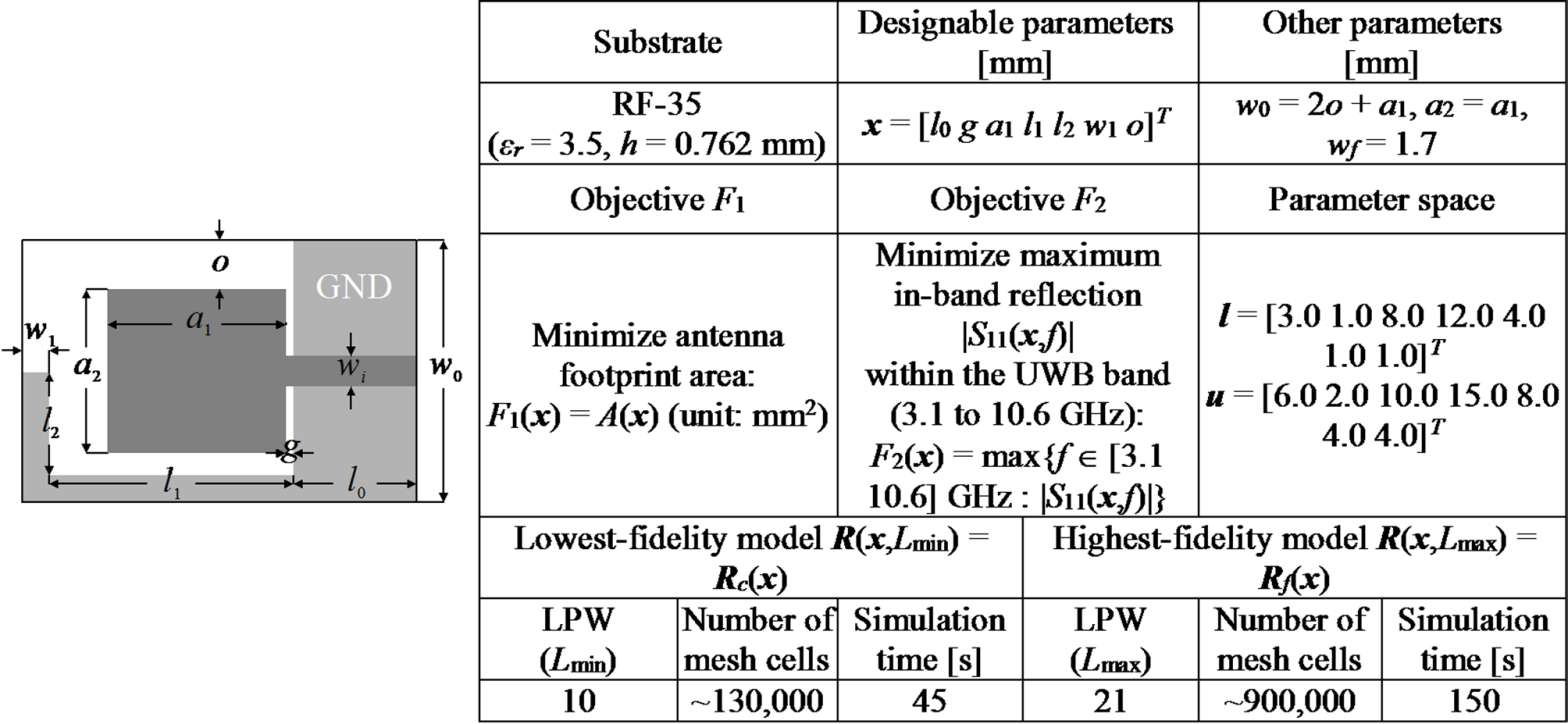
Antenna I^130^: Antenna structure, essential parameters, design objectives, and setup of variable-fidelity EM models.

**Fig. 8:**
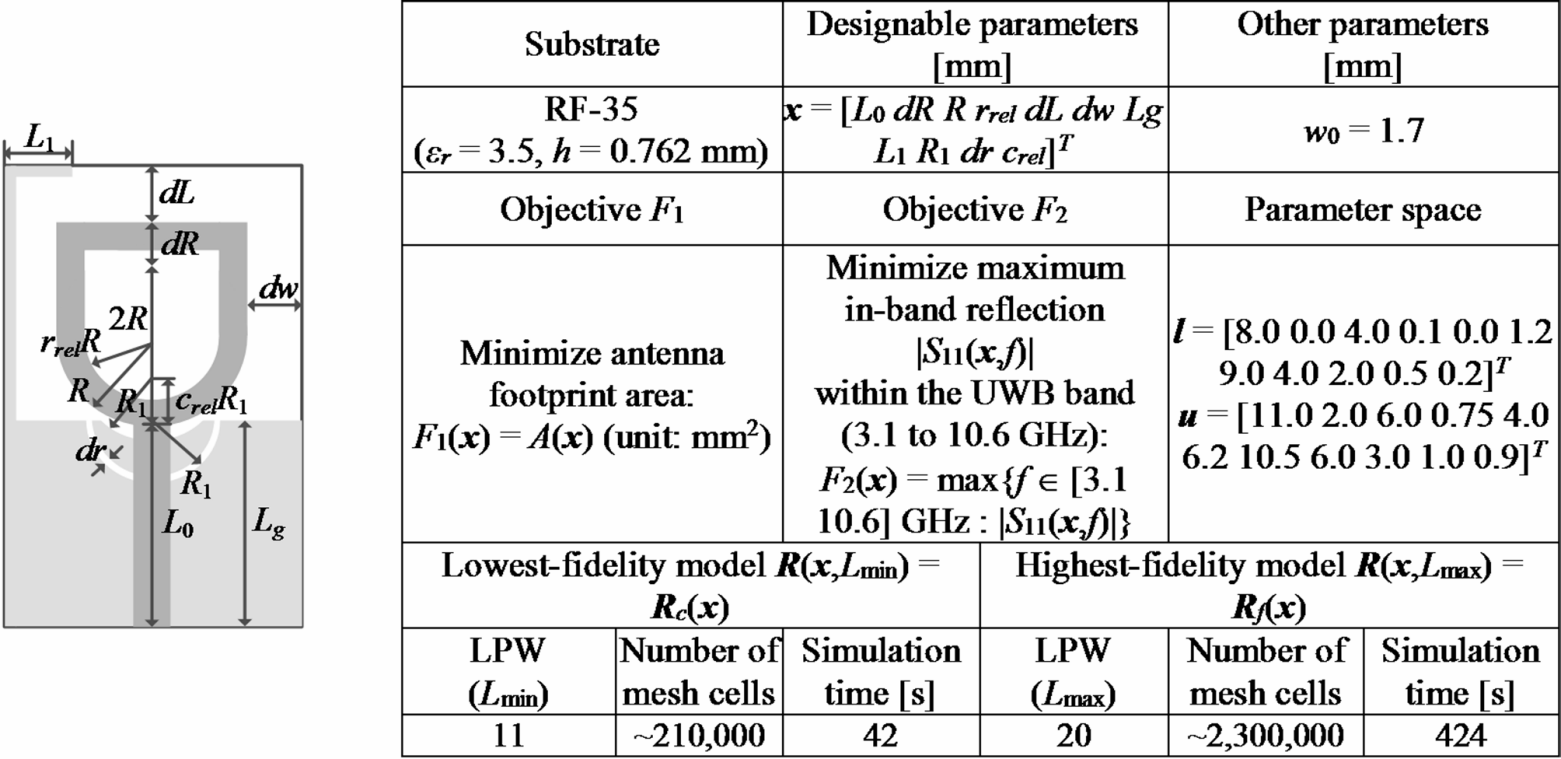
Antenna II^131^: Antenna structure, essential parameters, design objectives, and setup of variable-resolution EM models.

**Fig. 9:**
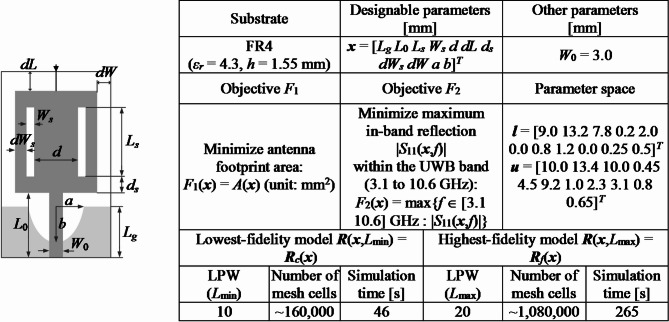
Antenna III^132^: Antenna structure, essential parameters, design objectives, and setup of variable-fidelity EM models.

**Fig. 10:**
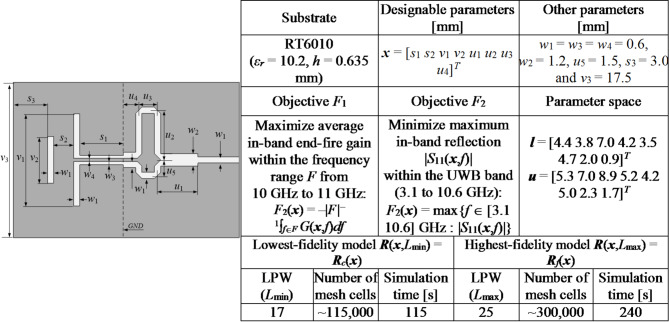
Antenna IV^133^: Antenna structure, essential parameters, design objectives, and setup of variable-fidelity EM models.

For verification purposes, we utilize four microstrip antennas depicted in Figs. 7, 8, 9 and 10, denoted as Antenna I, II, III, and IV, respectively. These include three broadband miniaturized monopoles and a quasi-Yagi antenna. The antenna structures utilized as verification examples were originally introduced in the following works: Antenna I^130^, Antenna II^131^, Antenna III^132^, and Antenna IV^133^, where all relevant details can be found. The first three antennas are compact monopoles operating within the ultra-wideband (UWB) from 3.1 GHz to 10.6 GHz. Antenna I uses a rectangular radiator and an L-shaped stub in the ground plane to lengthen the current patch, thereby facilitating impedance matching at the lower edge of the operating frequency range. Antenna II employs a semi-circular radiator with an inner slot, an L-shaped ground plane stub, and a defected ground below the feeding line, also to improve the impedance matching. Antenna III uses two rectangular slots in the rectangular radiator and an elliptical-shaped ground plane slot. Antenna IV is a quasi-Yagi structure with an integrated balun, operating within the target frequency range of 10 GHz to 11 GHz. For this structure, we are interested in impedance matching and end-fire gain, which is to be maximized.

Figures 7, 8, 9 and 10 provide also the substrate parameters and design variables for each antenna, as well as details about the electromagnetic models setup. The range of model fidelities has been established following the discussion in Sect. 2.2. All the simulations were performed on Intel Xeon 2.1 GHz dual-core CPU with 128 GB RAM. The simulation times reported in Figs. 7, 8, 9 and 10 correspond to this hardware configuration. Notably, the simulation time ratio between high fidelity (***R***_*f*_) and low fidelity (***R***_*c*_) varies significantly from around two for Antenna IV to over ten for Antenna II. This suggests potentially significant computational advantages resulting from the incorporation of variable-fidelity EM analysis.

An alternative approach to reducing the computational cost of simulation-based design is to run the design procedure on a GPU. Yet, GPU usage would only lower the portion of the computational cost associated with the EM simulations, not the training of NN models, which we employ for modeling antenna response. This is because the architectures of the NN models used in this work are relatively simple. The cost of training these models is negligible in our overall computational budget and is therefore omitted from consideration.

Furthermore, the potential reduction in EM simulation time from GPU acceleration would be limited, as the antenna structures used for verification are of moderate complexity (with simulation models ranging from approximately 100,000 to 2,000,000 cells). Consequently, the use of a GPU would be expected to reduce the total EM simulation cost by only about 30–50%. This is primarily due to the fact that a significant portion of the total simulation time is spent on structure discretization and preparatory steps, while the FDTD simulation itself accounts for less than half of the total simulation cost.

In addition, Figs. 7, 8, 9 and 10 present details pertaining to the parameter spaces, denoted by the lower and upper bound vectors (***l*** and ***u***, respectively), as well as the design objectives. For Antennas I, II, and III, the objectives include reducing the antenna footprint area (*F*_1_) and minimizing the maximum in-band reflection level (*F*_2_). In the case of Antenna IV, the primary objective is to maximize the average in-band end-fire gain (*F*_1_), while the secondary goal is minimizing the maximum in-band reflection (*F*_2_). Two-objective test problems are predominantly considered to facilitate the visualization of the results. In three cases (Antennas I through III), the design objective related to the electrical characteristics of the antenna pertains to its reflection response. In the case of Antenna IV, both the reflection characteristics and the end-fire gain are considered. Nevertheless, the proposed multi-objective optimization framework can also be applied to the optimization of other performance figures, such as the radiation pattern or directivity.

### Experimental setup

The verification antennas, introduced in Sect. 3.1, undergo optimization using the proposed variable-fidelity machine learning framework with ANN surrogate models. In all test cases, the default control parameters are used (see Table 3). The ultimate result of the optimization process is a representation of the Pareto front, which constitutes a subset of all non-dominated parameter vectors within the most recent EM dataset {***x***_*T*_^(*i.j*)^}.

The presented methodology has been compared to several surrogate-assisted MO techniques outlined in Table 4. The first two algorithms are single-shot procedures, with the surrogate built up front, and optimized using MOEA afterwards. In Algorithm 1, the underlying surrogate model is a kriging interpolant; for Algorithm 2, it is ANN (multi-layer perceptron). Both Algorithms 1 and 2 are executed using different numbers of data samples: 400 and 1600. Algorithm 3 is essentially a single-fidelity counterpart of the proposed method, where MO is performed utilizing one (high-fidelity) EM model. Also, the dataset updating strategy is straightforward, as each infill point is automatically retained (no need of removing any samples). The reason for including this method is to verify the computational benefits of employing multi-fidelity computational models.

**Table 4:** Surrogate-assisted algorithms used to benchmark the proposed MO procedure with variable-fidelity machine-learning.

Algorithm	General information	Surrogate model	Characterization
1	One-shot surrogate-assisted MO procedure	Kriging interpolation (Gaussian correlation functions and first-order polynomial as a trend function^84^)	Surrogate constructed using *N*_*S*_ data samples, then optimized using MOEA; Selected non-dominated samples evaluated with EM analysis to form the final outcome of the algorithm
2		Neural network (setup: multi- layer perceptron, two hidden layers with ten neurons each; training: Levenberg-Marquardt algorithm)	
3	Machine learning algorithm with ANN surrogates	ANN surrogates: Initial sampling and surrogate model setup the same as discussed in section “Initial sampling. ANN regression models”; Infill point generation as discussed in sections “Multi-objective evolutionary algorithm” and “Infill point generation” (surrogate optimization using MOEA); EM-simulation dataset updates by adding all infill points to the existing dataset	Search process executed using a single (high-fidelity) EM simulation model

### Numerical results

**Fig. 11:**
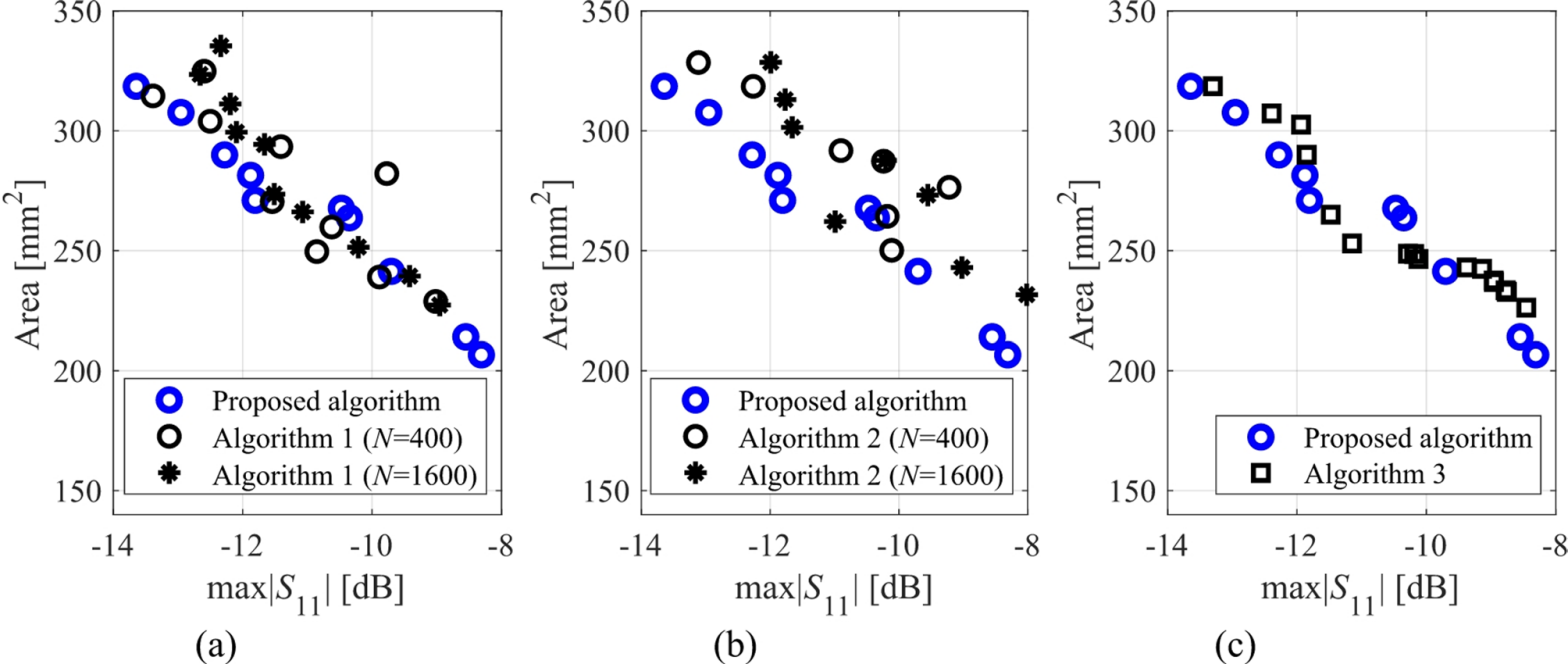
Pareto sets for Antenna I generated by the introduced MO procedure and the benchmark algorithms: (a) proposed algorithm versus Algorithm 3, (b) proposed algorithm versus Algorithm 1, (c) proposed algorithm versus Algorithm 2.

**Fig. 12:**
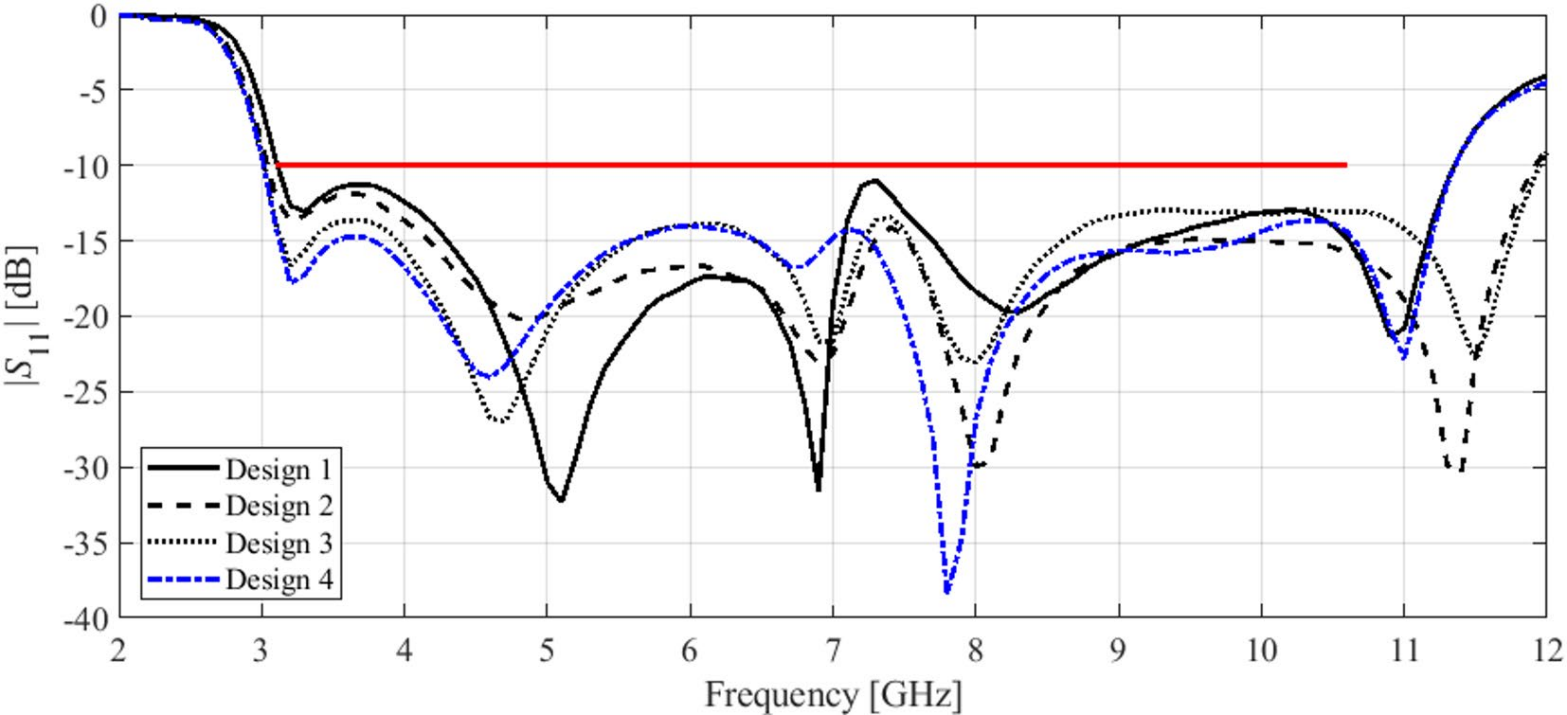
Reflection characteristics of Antenna I at exemplary Pareto-optimal designs: (a) Design 1 (*A* = 241 mm^2^), (b) Design 2 (*A* = 271 mm^2^), (c) Design 3 (*A* = 308 mm^2^), (d) Design 4 (*A* = 319 mm^2^). The intended range of operating frequency marked by horizontal line.

**Fig. 13:**
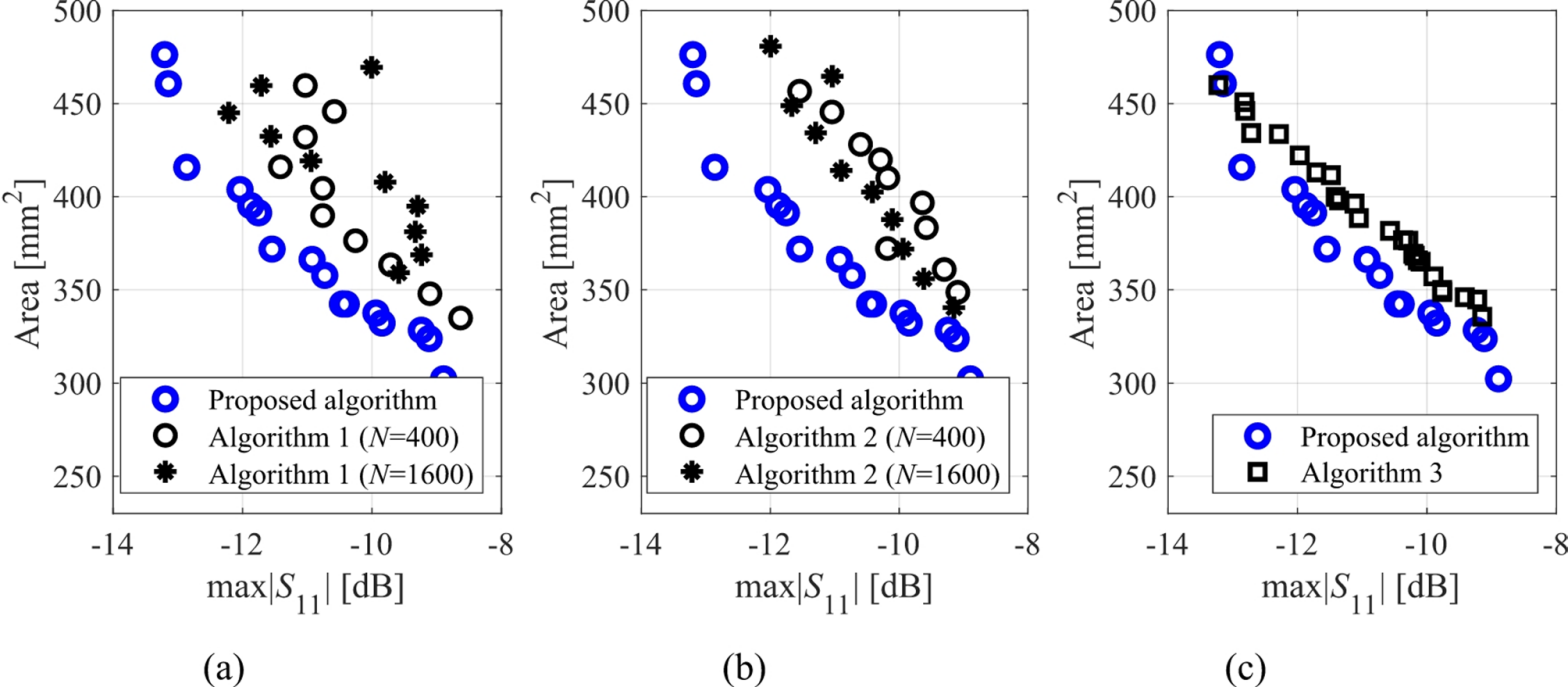
Pareto sets for Antenna II generated by the introduced MO procedure and the benchmark algorithms: (a) proposed algorithm versus Algorithm 3, (b) proposed algorithm versus Algorithm 1, (c) proposed algorithm versus Algorithm 2.

**Fig. 14:**
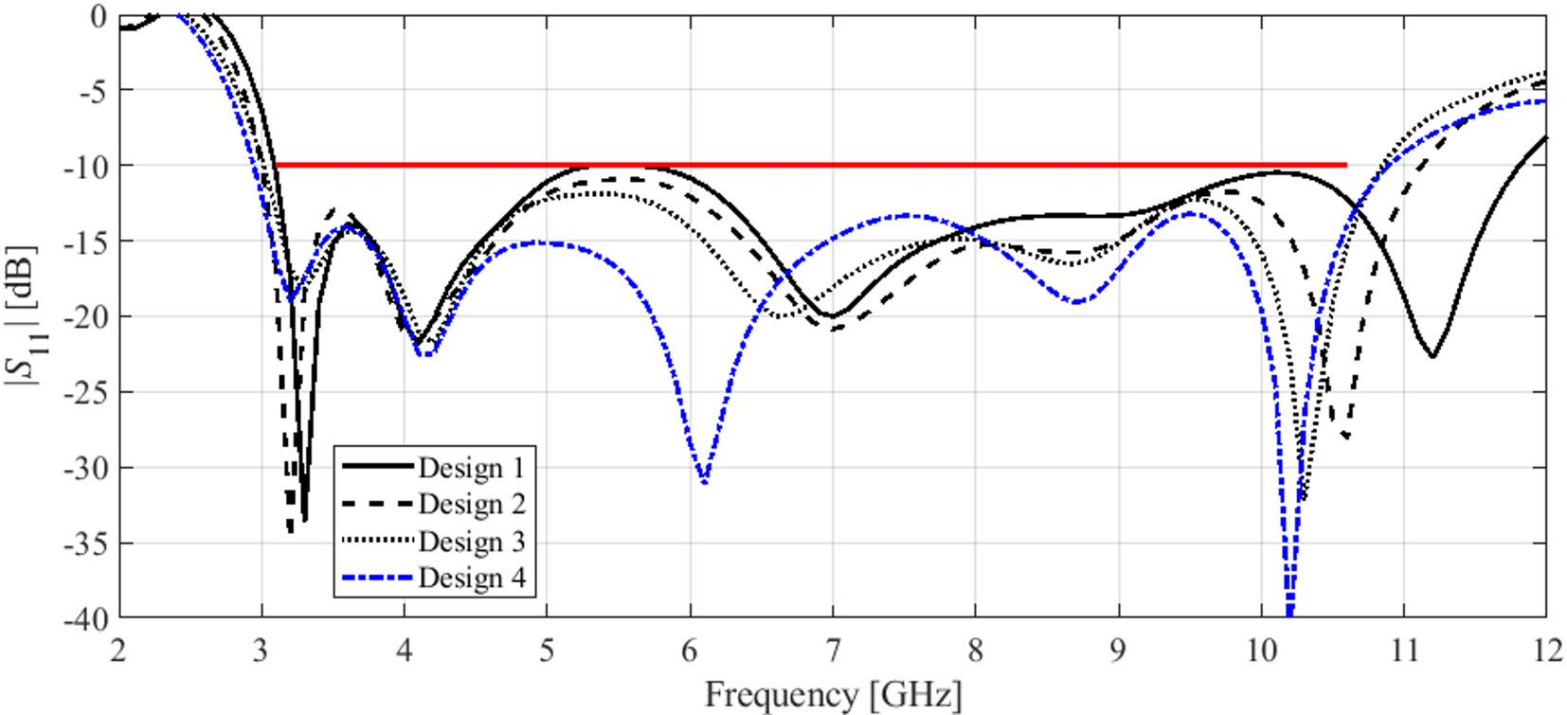
Reflection characteristics of Antenna II at exemplary Pareto-optimal designs: (a) Design 1 (*A* = 337 mm^2^), (b) Design 2 (*A* = 366 mm^2^), (c) Design 3 (*A* = 395 mm^2^), (d) Design 4 (*A* = 476 mm^2^). The intended range of operating frequency marked by horizontal line.

**Fig. 15:**
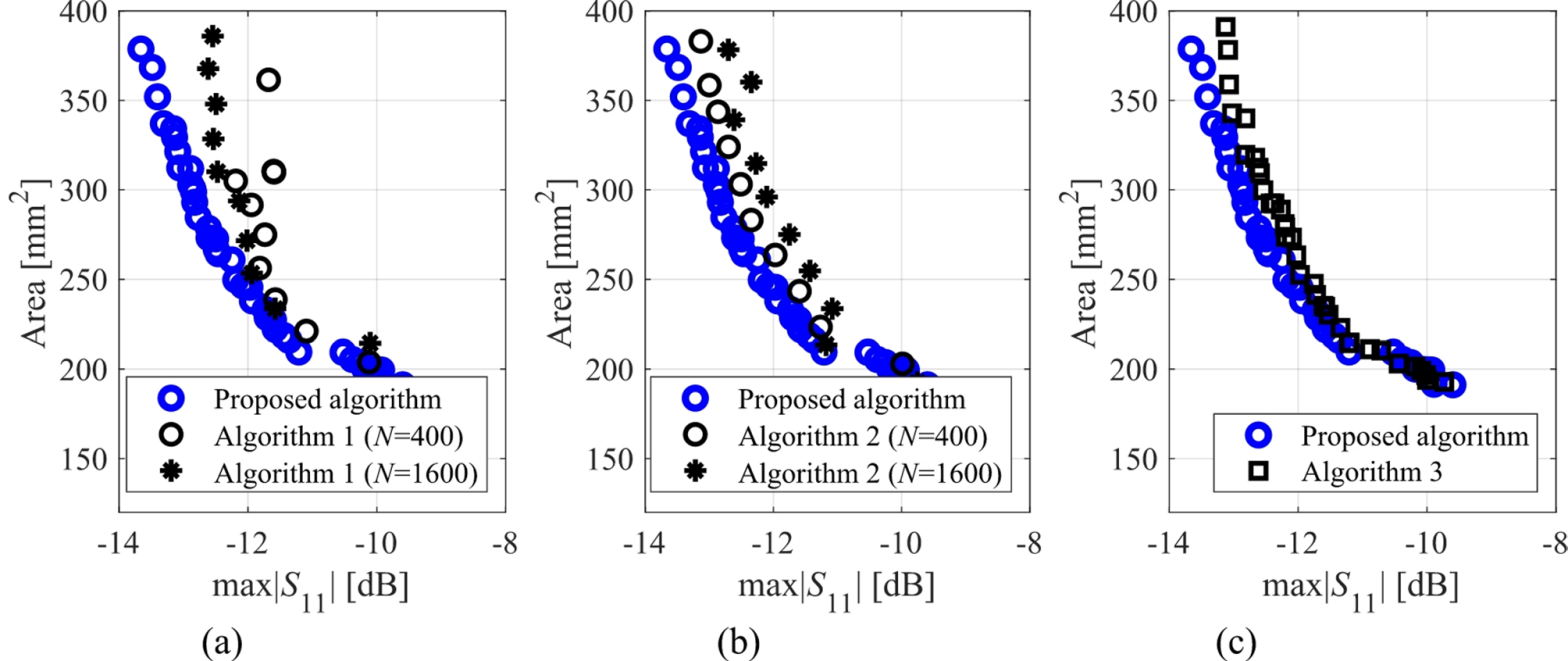
Pareto sets for Antenna III generated by the introduced MO procedure and the benchmark algorithms: (a) proposed algorithm versus Algorithm 3, (b) proposed algorithm versus Algorithm 1, (c) proposed algorithm versus Algorithm 2.

**Fig. 16:**
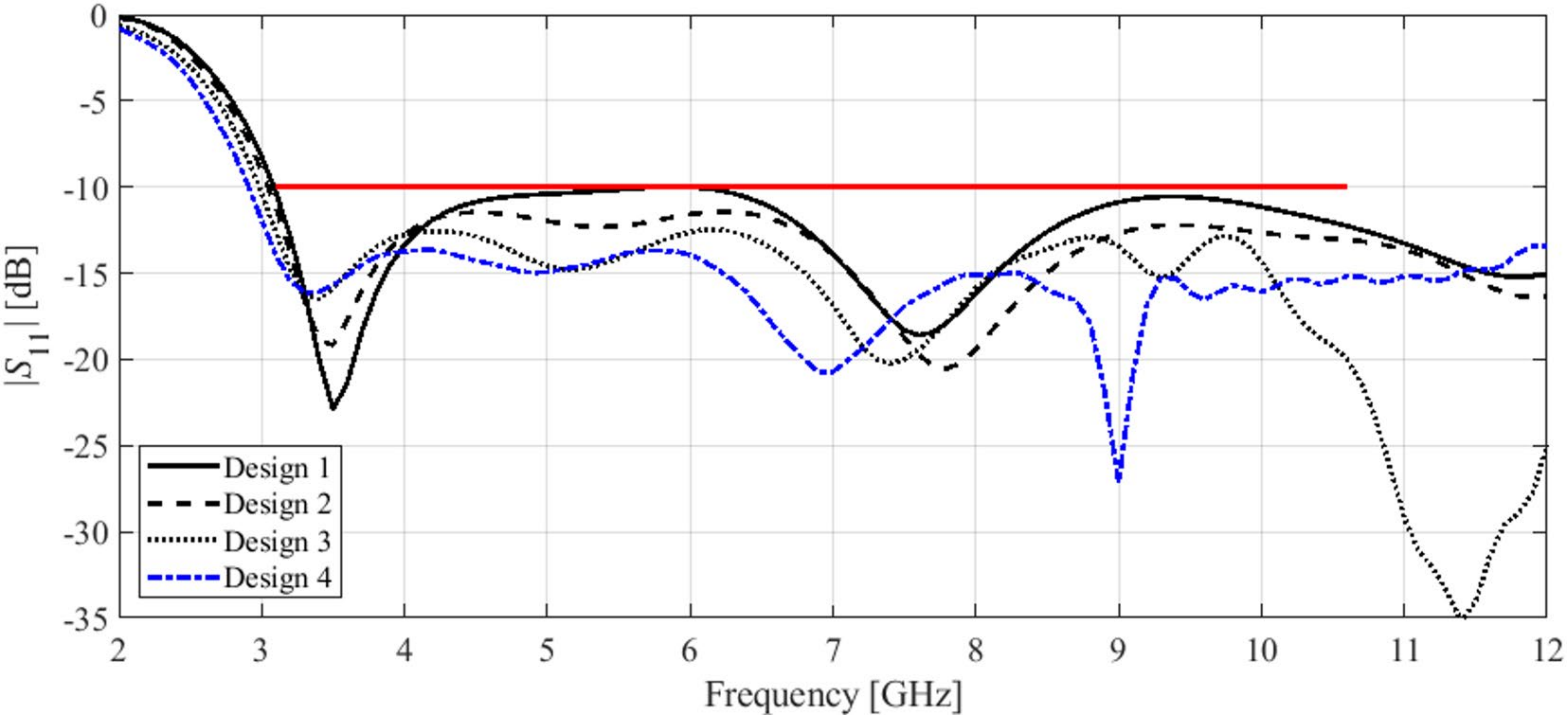
Reflection characteristics of Antenna III at exemplary Pareto-optimal designs: (a) Design 1 (*A* = 200 mm^2^), (b) Design 2 (*A* = 210 mm^2^), (c) Design 3 (*A* = 264 mm^2^), (d) Design 4 (*A* = 379 mm^2^). The intended range of operating frequency marked by horizontal line.

**Fig. 17:**
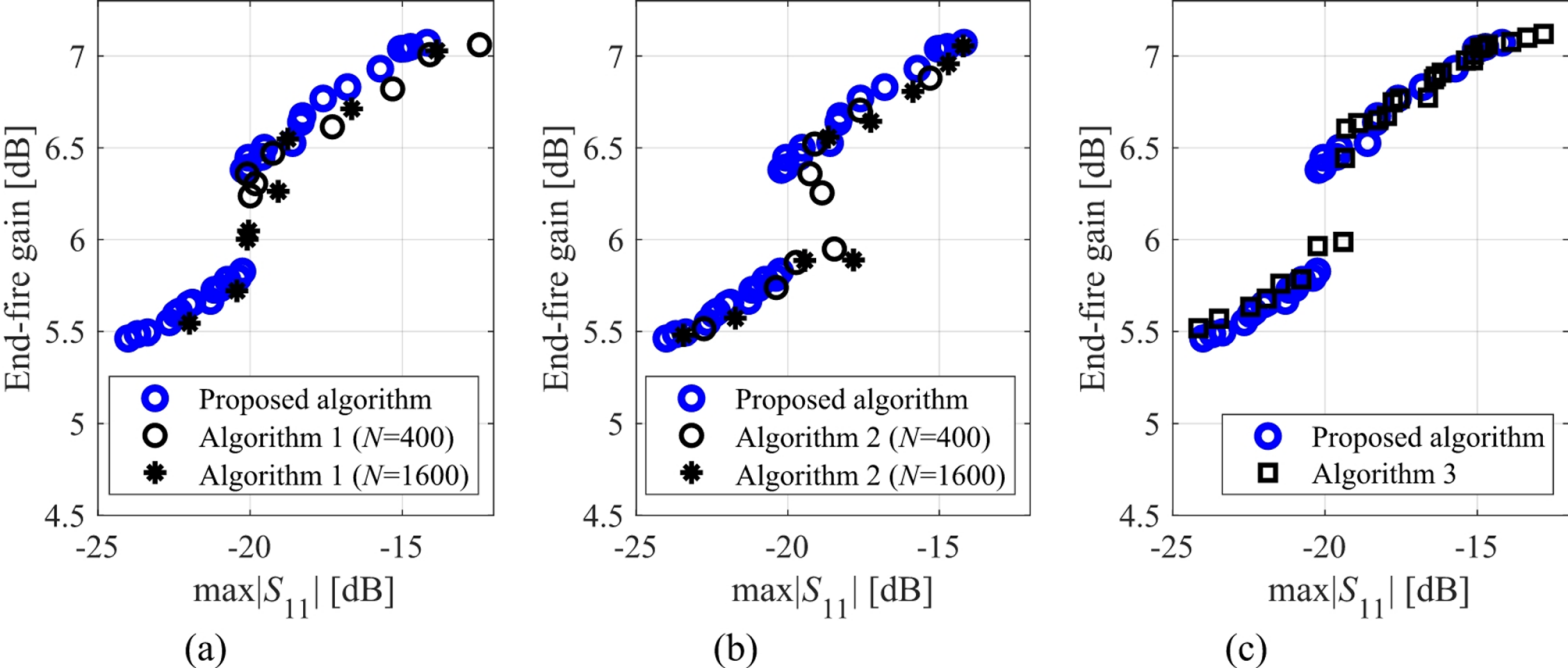
Pareto sets for Antenna IV generated by the introduced MO procedure and the benchmark algorithms: (a) proposed algorithm versus Algorithm 3, (b) proposed algorithm versus Algorithm 1, (c) proposed algorithm versus Algorithm 2.

**Fig. 18:**
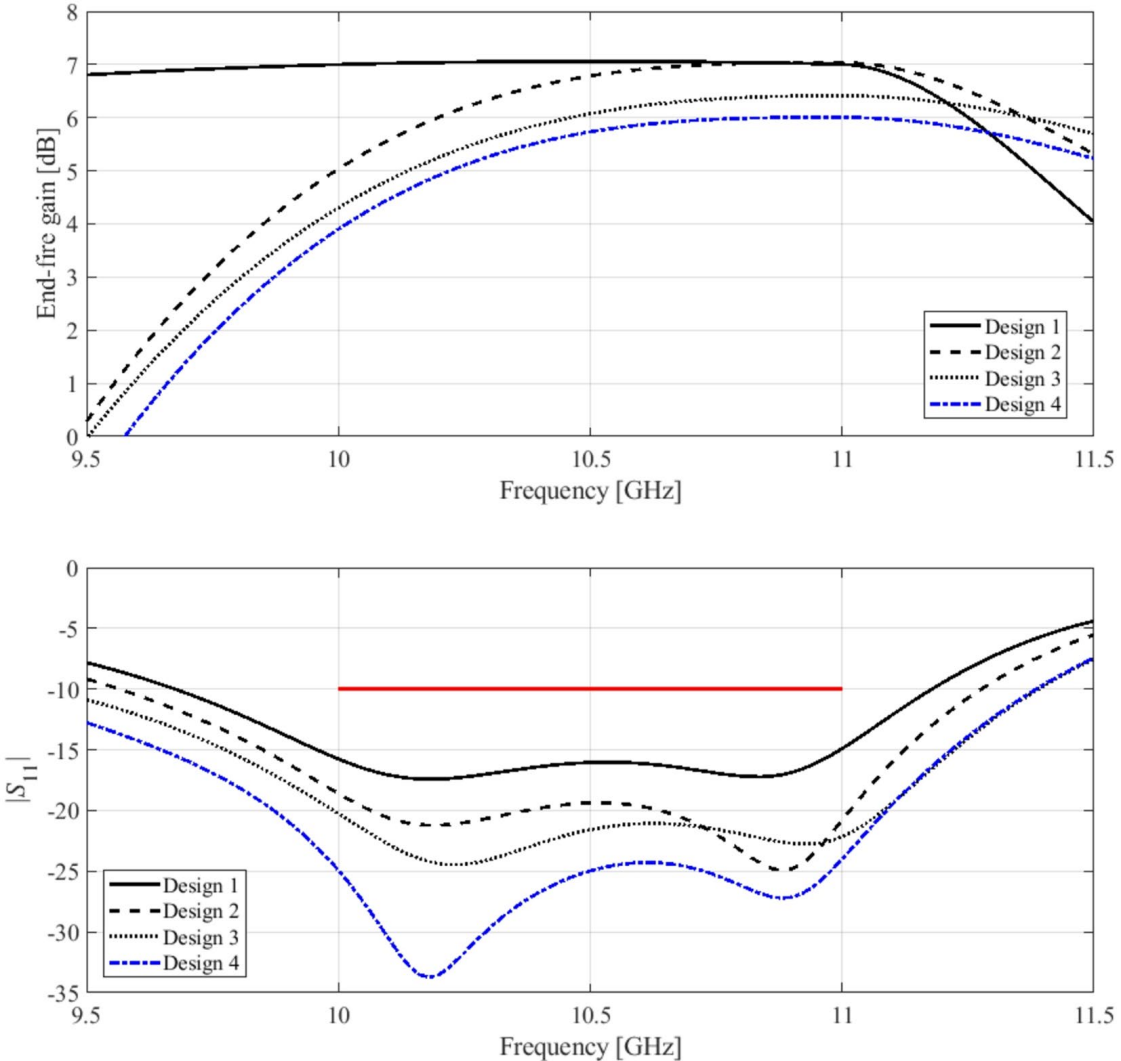
Reflection (black) and end-fire gain (gray) response of Antenna IV at exemplary Pareto-optimal designs: (a) Design 1 (average gain 7.1 dB), (b) Design 1 (average gain 6.5 dB), (c) Design 2 (average gain 5.8 dB), (d) Design 3 (average gain 5.5 dB). The intended range of operating frequency marked by horizontal line.

Here, we put together the outcome of the presented optimization framework and the results generated with the benchmark methods (Figs. 11, 12, 13, 14, 15, 16, 17 and 18). Figures 11, 13 and 15, and 17 show a comparison between the Pareto set obtained using our technique and those generated by Algorithms 1, 2, and 3, for Antennas I, II, III, and IV, respectively. Figures 12, 14 and 16, and 18 show antenna characteristics at some Pareto-optimal solutions generated by the proposed method. High-fidelity EM analysis is utilized to evaluate both antenna characteristics and the design objectives. Table 5 gathers data concerning the CPU cost of the MO process. The cost only includes the expenses associated with full-wave electromagnetic analysis of the antenna structures. Whereas the costs entailed by metamodel training and execution of MOEA are neglected because—for the considered cases—their contribution to the overall running time of the algorithm is minor. For example, a typical ANN training time is about twenty seconds, whereas high-fidelity EM analysis (single simulation) varies between three and seven minutes. The total cost of the proposed procedure provided in Table 5 includes the cost of initial sampling, which is performed entirely using low-fidelity simulations. Observe that for our method, the cost is expressed in terms of the equivalent number of high-fidelity EM simulations, which accounts for the time evaluation ratio between the high- and lower-fidelity models. For all considered cases, the number of initial sample points used to construct the first ANN surrogate is 100 (see Table 3).

**Table 5:** Multi-objective optimization using the introduced procedure and the benchmark algorithms: cost breakdown.

Algorithm		Optimization cost^#^ and hypervolume^@^			
		Antenna I	Antenna II	Antenna III	Antenna IV
This work (variable-fidelity ML with ANN surrogates)		150.1 [0.65]	150.4 [0.67]	252.5 [0.77]	264.4 [0.67]
Algorithm 1	*N* = 400	400 [0.55]	400 [0.43]	400 [0.63]	400 [0.59]
	*N* = 1600	1600 [0.58]	1600 [0.40]	1600 [0.65]	1600 [0.58]
Algorithm 2	*N* = 400	400 [0.50]	400 [0.42]	400 [0.67]	400 [0.54]
	*N* = 1600	1600 [0.55]	1600 [0.44]	1600 [0.68]	1600 [0.55]
Algorithm 3		390 [0.60]	320 [0.61]	330 [0.76]	340 [0.66]

^#^ The cost of benchmark algorithms is equivalent to the total number of EM simulations performed. For the proposed method, the cost is expressed in terms of the equivalent number of high-fidelity EM simulations by taking into account the time evaluation ratio between the high- and lower-fidelity models.

^$^Hypervolume, provided in square brackets, is computed for normalized objectives.

The proposed optimization technique is compared to benchmark methods with respect to two criteria: (i) computational cost, and (ii) reliability. The first one is straightforward to measure using the number of high-fidelity EM simulations, which ensures a fair comparison: the cheaper method is better in terms of this criterion, cf. Table 5. Regarding the second criterion, we only use visual inspection of the Pareto sets shown in the respective figures (Figs. 11, 13 and 15, and 17, for Antennas I, II, III, and IV). It is clear that the Pareto sets produced by the proposed approach are distinctly better than the sets rendered by the benchmark techniques, so no numerical metrics are necessary.

**Fig. 19:**
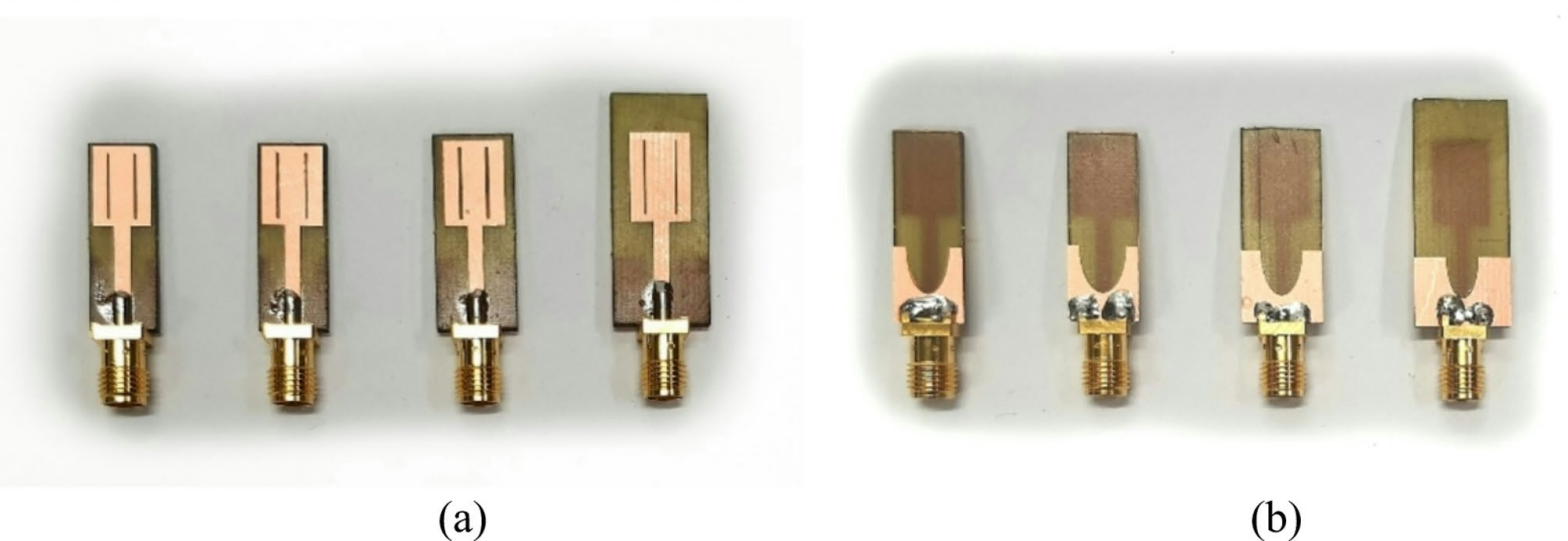
Photographs of prototypes representing Pareto-optimal designs of Antenna III generated by the introduced procedure (Designs 1 through 4 of Fig. 16): (a) front views, (b) back views.

**Fig. 20:**
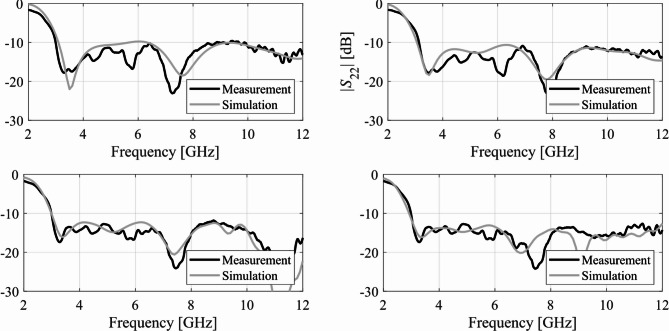
Measurements and EM-simulations at the Pareto-optimal designs of Antenna III rendered by the introduced approach: (a) Design 1, (b) Design 2, (c) Design 3, (d) Design 4.

**Fig. 21:**
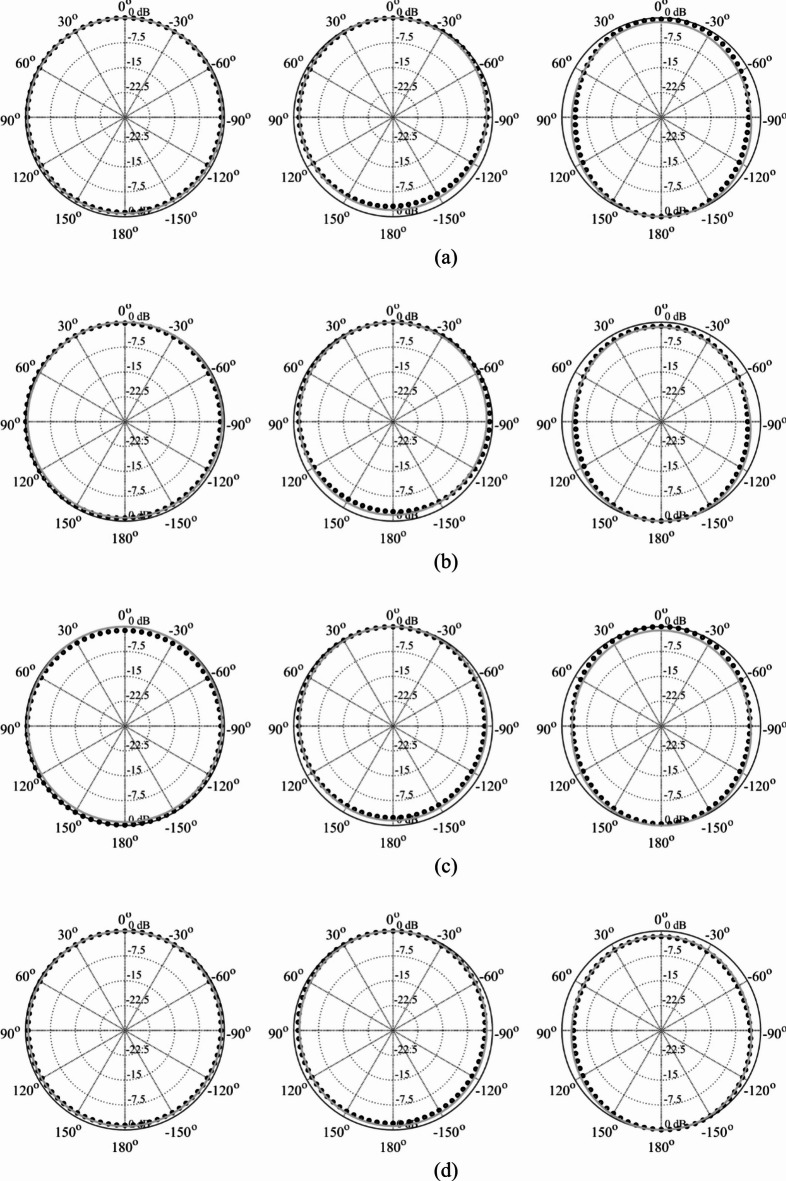
H-plane radiation patterns: simulations (grey) and measurements (black) for 4 GHz (left), 6 GHz (middle), and 8 GHz (right) of Pareto-optimal solutions of Antenna III generated by the introduced approach: (a) Design 1, (b) Design 2, (c) Design 3, (d) Design 4.

**Fig. 22:**
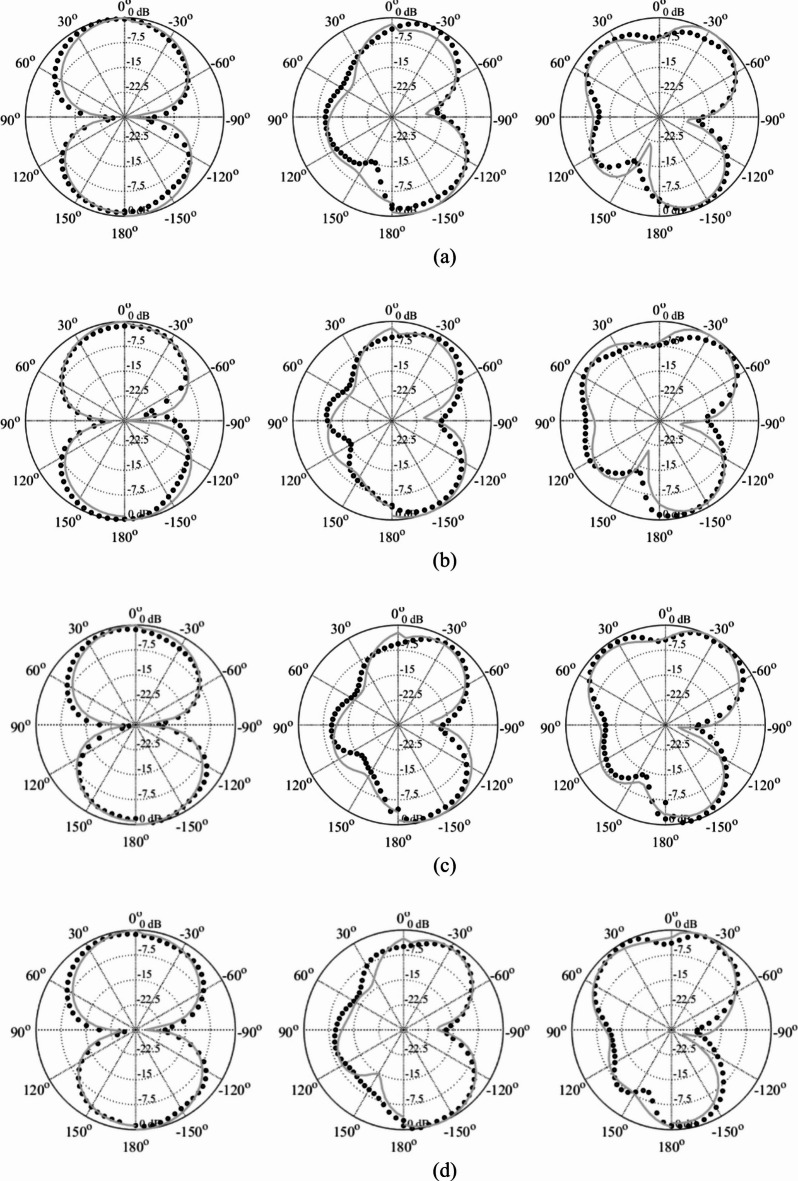
E-plane radiation patterns: simulations (grey) and measurements (black) for 4 GHz (left), 6 GHz (middle), and 8 GHz (right) of Pareto-optimal solutions of Antenna III generated by the introduced approach: (a) Design 1, (b) Design 2, (c) Design 3, (d) Design 4.

As a supplementary demonstration, the Pareto-optimal designs of Antenna III, presented in Fig. 15 (Designs 1 through 4), were fabricated and experimentally validated. Table 6 gathers the values of the geometry parameters corresponding to Designs 1 through 4. The antenna prototypes are depicted in Fig. 19, while the measured and simulated reflection coefficients, as well as the H-plane and E-plane radiation patterns, are shown in Figs. 20, 21 and 22, respectively. The measurement setup includes a double-rigged horn antenna (Geozondas GZ0226DRH) and Anritsu VNA (MS4644B). Notably, the correspondence between the EM simulations and measurements is excellent. Furthermore, the reduction in antenna size does not adversely affect radiation properties.

**Table 6:** Antenna III: values of design parameters for Pareto-optimal designs of Fig. 19.

Parameter	L_g_	L_0_	L_s_	W_s_	d	dL	d_s_	dW_s_	dW	a	b
Design 1	9.53	13.28	8.38	0.37	2.97	0.42	0.89	1.69	0.64	0.63	0.61
Design 2	9.70	13.32	8.39	0.30	3.29	0.41	0.92	1.96	0.47	0.67	0.62
Design 3	9.43	13.33	9.16	0.40	3.09	0.90	0.92	1.99	1.30	0.52	0.62
Design 1	9.18	13.35	9.64	0.29	3.80	5.16	0.90	1.75	2.39	0.34	0.59

### Discussion

The results reported in Sect. 3.3 demonstrate a competitive level of performance of the proposed algorithm in terms of dependability and computational efficiency. As shown in Figs. 12, 14 and 16, and 18, the Pareto sets’ quality ensured by our framework considerably exceeds those obtained using Algorithms 1 and 2. The reason is the limited accuracy of both the kriging and ANN metamodels. For *N* = 400 training points, the average relative RMS error of those surrogates is about 20% for Antennas I and II, and about six to eight% for Antenna III and IV.

When the models are constructed using *N* = 1600 data samples, the errors go down to about 15% for Antennas I and II, and to between four and six% for Antennas III and IV. This inaccuracy is reflected in a large distance between the Pareto-optimal designs generated by our method and EM-simulated solutions found by Algorithms 1 and 2. The distance is smaller for Antennas III and IV, which is indicative of the importance of the good predictive power of the metamodels. Whereas the alignment between the Pareto sets rendered by the benchmark Algorithm 3 and those generated by the proposed framework is significantly better, which corroborates that the ML algorithms are capable of focusing the optimization process in the most encouraging subsets of the design space and enhance the surrogate by allocating the majority of infill points there.

The examination of computational costs presented in Table 5 reveals that the proposed algorithm, on average, necessitates approximately 200 high-fidelity EM simulations to complete the optimization process. Simultaneously, the costs associated with benchmark Algorithm 3, which is similar to the proposed technique except that it exclusively uses the high-fidelity EM model, are considerably higher (about 350 EM simulations on average). Thus, the incorporation of variable-fidelity computational models leads to 40% speedup. When comparing these numbers with Algorithms 1 and 2 (version with 1600-sample budget), the relative savings offered by our methodology are almost 90% while ensuring significantly higher Pareto front quality.

The assessment of the proposed MO surrogate-based optimization framework has been carried out based on the outcomes presented and discussed in Sect. 3.3. Below, a comparative analysis taking into account factors such as computational efficiency, Pareto front quality, and algorithm implementation is outlined.

- *Pareto front quality*. Our methodology renders Pareto fronts of a considerably better quality than the stat-of-the-art surrogate-assisted procedures (both using kriging and ANN surrogates) even when the latter utilize four-fold larger training data sets. The sole benchmark procedure capable of yielding similar quality of the Pareto front is the ML procedure operating exclusively on a high-fidelity model. However, its computational expenses are significantly higher.
- *Computational efficacy*. The average total optimization cost of our algorithm is remarkably low and equals to around two hundred fine EM analyses. These expenses are over 40% lower than that of the counterpart ML procedure using the high-fidelity EM model, which corroborates the adequacy of employing variable-fidelity simulations for cost-efficacy enhancement. As for the higher-budget one-shot surrogate-assisted procedures, the speedup of our algorithm is even more pronounced and reaches up to 90%. At the same time, Pareto front quality rendered using our framework not only did not deteriorate but even improved.
- *Setup*. The setup of our algorithm is simple, with only a few control parameters (mainly pertaining to dataset sizes and model fidelity transition). The recommended values of these parameters have been employed to solve MO design tasks for various design objectives and for different antenna structures of varying dimensionalities. This indicates that the control parameters of our framework do not have to be tuned to the specific case problems.

Overall, the performance of the proposed MO approach qualifies it a feasible option to existing machine learning methodologies. Low running cost and dependability are the two most important features, with structural simplicity, easy implementation, and straightforward setup being other attractive properties, especially in the light of practical design utility. Moreover, it should be reiterated that the verification problems considered in this work are significantly more challenging than those typically used to demonstrate surrogate-assisted multi-objective antenna optimization procedures. Ultimately, the conducted comparative studies are considerably more extensive than what is typically reported in the literature. This pertains not only to the number of considered test cases and their diversity but also to the number of benchmark methods.

## Conclusion

This article proposed an innovative approach to rapid and dependable multi-objective optimization of antenna systems. Our algorithm is a surrogate-assisted machine learning procedure, which employs the ANNs as the principal metamodeling approach. The Pareto solutions are created by optimizing the ANN metamodel using MOEA algorithm. The termination condition is determined by comparing the distance between EM-evaluated Pareto sets found in subsequent iterations. Further reduction of computational expenses is achieved by utilizing multi-fidelity simulations models, with the model resolution gradually increasing during the algorithm run.

The presented optimization strategy presented has been validated using four microstrip antennas and compared to several benchmark methodologies involving surrogate models. The results obtained affirm the competitive performance of our algorithm, demonstrating both reliability and computational efficiency. The average running cost corresponds to only about two hundred high-fidelity antenna simulations. The relative savings over one-shot approaches are almost 90%. The acceleration over a machine learning procedure involving ANN metamodels but single-fidelity EM simulations is as high as 40%. At the same time, our technique yields the Pareto front of the highest quality. The proposed MO framework is versatile and has been demonstrated to generate consistent results for a broad range of test cases of different dimensionalities (from seven to eleven) and defined over large parameter spaces. One of the attractive features of the discussed method is a simple setup and a reduced number of mostly problem-independent control parameters. An essential goal of future research endeavors will be further improvement of the efficacy of the presented technique, among others, by incorporating dimensionality reduction approaches, as well as demonstration of its suitability for test problems with a higher number of design objectives.

## Data Availability

Data availability: The datasets generated during and/or analysed during the current study are available from the corresponding author on reasonable request. Contact person: anna.dabrowska@pg.edu.pl.
